# Mg(H_2_O)_2_[TeO_2_(OH)_4_]: a polytypic structure with a two-mode disordered stacking arrangement

**DOI:** 10.1107/S2052520621006223

**Published:** 2021-07-23

**Authors:** Berthold Stöger, Hannes Krüger, Matthias Weil

**Affiliations:** a X-Ray Center, TU Wien, Getreidemarkt 9, 1060 Vienna, Austria; b Institute of Mineralogy and Petrography, University of Innsbruck, Innrain 52, 1060 Innsbruck, Austria; c Institute of Chemical Technologies and Analytics, TU Wien, Getreidemarkt 9/164-SC, 1060 Vienna, Austria

**Keywords:** polytypism, order–disorder (OD) theory, disorder, diffuse scattering, homometry

## Abstract

Crystals of the hydrous magnesium orthotellurate(VI) Mg(H_2_O)_2_[TeO_2_(OH)_4_] were grown by slow diffusion of an aqueous MgCl_2_ solution into a KOH/Te(OH)_6_ solution immobilized in gelatin. The crystal structure is built of sheets of nearly regular corner-sharing [MgO_6_] and [TeO_6_] octahedra. Half of the bridging O atoms are connected to disordered H atoms, which are located in rhomboidal voids (long and short diameters of ∼5.0 and ∼2.5 Å, respectively) of these layers. Moreover, the Te^VI^ atom connects to two OH^−^ ions and the Mg^II^ atom to two H_2_O molecules. The OH^−^ ions and H_2_O molecules connect adjacent layers forming a disordered hydrogen-bonding network. In a given layer, an adjacent layer may be positioned in four ways, which can be characterized by one of two origin shifts and one of two orientations with respect to [100]. The crystals feature a disordered stacking arrangement, leading to rods of diffuse scattering in the diffraction pattern. The polytypism is explained by application of the order–disorder (OD) theory. Different refinement models are compared and the diffuse scattering is evaluated with structure factor calculations. The correlation coefficient of subsequent origin shifts is ∼ −0.33, whereas the orientation of the layers is essentially random. Determining the latter is particularly difficult owing to a small contribution to the diffraction pattern and virtually indistinguishable diffraction patterns for pairs of correlations with the same absolute value. On longer standing in a glass vial, an ordered polytype forms.

## Abstract 2

Crystals of the polytypic Mg(H_2_O)_2_[TeO_2_(OH)_4_] feature one-dimensional diffuse scattering owing to correlated disorder.

## 1. Introduction   

Orthotellurates(VI) of alkaline earth metals with general formula *M*_2_ *M*′[Te^VI^O_6_] bear interesting crystal-chemical and physico-chemical aspects, and a number of these phases and their solid solutions are structurally well characterized (Prior *et al.*, 2005 ▸; Fu *et al.*, 2008 ▸). The structures of nearly all alkaline earth metal tellurates (except Be) with a single *M* ^II^ cation and the general formula 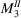 [Te^VI^O_6_] have been elucidated [*M* = Mg: Schulz & Bayer (1971 ▸); *M* = Ca: Hottentot & Loopstra (1981 ▸); *M* = Sr, Ba: Stöger *et al.* (2010 ▸)]. The structures of these tellurates are characterized by rigid, practically regular, octahedral [TeO_6_]^6−^ units. Ca_3_[TeO_6_] (*P*2_1_/*n*, *Z* = 2), Sr_3_[TeO_6_] ( 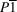 , *Z* = 32) and Ba_3_[TeO_6_] (*I*4_1_/*a*, *Z* = 80) are hettotypes of the double perovskite structure type, where the *M* ^II^ atom occupies two positions with distinctly different coordination spheres. The ionic radius of Mg^II^, on the other hand, is incompatible with the large voids required by the double perovskite aristotype and therefore Mg_3_[TeO_6_] ( 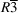 , *Z* = 2) crystallizes in a different structure type, isotypic with Mn_3_[TeO_6_] (Weil, 2006 ▸).

During our ongoing studies of hydrous derivatives of *M*_3_[TeO_6_] phases with *M* = Mg, Ca, Sr, Ba we obtained single crystals of the title compound, Mg(H_2_O)_2_[TeO_2_(OH)_4_], with a unique crystal structure. So far, hydrous alkaline earth tellurates have only been described for Ba (Weil *et al.*, 2016 ▸). We report here on the structure determination and description of the polytypic structure as well as on thermal behavior of Mg(H_2_O)_2_[TeO_2_(OH)_4_].

Symbols used are summarized in Appendix *A* [App appa].

## 2. Experimental   

### 2.1. Synthesis and crystal growth   

Crystals of Mg(H_2_O)_2_[TeO_2_(OH)_4_] were grown in gelatin using a gel diffusion technique (Heinisch, 1996 ▸). Three gelatin sheets (∼4.5 g) were dissolved in a solution of KOH (4.34 g, 85%wt) and Te(OH)_6_ (7.89 g) in water (300 ml). From this mixture, 25 ml of the solution were introduced into a large test tube. After solidification, the gel was covered with 10 ml of a neutral gelatin solution, prepared by dissolving one gelatin sheet (∼1.5 g) in water (100 ml). After solidification of the second gelatin layer, it was covered with Mg^II^ solution (10 ml, 0.5%wt) which was obtained by dissolving MgCl_2_·6H_2_O (4.16 g) in water (100 ml). The test tube was sealed with wrapping film and kept at 295 K for one month. Square-bipyramidal crystals of Mg(H_2_O)_2_[TeO_2_(OH)_4_] had formed at the interface of both gelatin layers. The gel was cut with a scalpel and crystals with an adequate size for single-crystal diffraction were isolated under a polarizing microscope.

### 2.2. Data collection   

Diffraction intensities for structure refinements were collected at room temperature using fine-sliced ω- and φ-scans on a Bruker KAPPA APEX II diffractometer equipped with a CCD camera (Mo 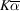 radiation, graphite-monochromated). Bragg intensities were reduced using the *SAINT-Plus* software (Bruker, 2017 ▸). An absorption correction was applied using a multi-scan approach with *SADABS* (Bruker, 2017 ▸) using the 4/*mmm* Laue group.

Inspection of the diffraction pattern (reconstructed reciprocal space layers) revealed lines with pronounced diffuse scattering. For the quantitative analysis of the diffuse scattering, a second crystal was measured with special attention paid to minimization of artifacts on a Stoe IPDS-II image-plate diffractometer using graphite-monochromated Mo 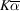 radiation produced by a conventional sealed X-ray tube operated at 50 kV and 40 mA. A 0.5 mm fiber optic collimator and beam stop were positioned in such a way that the free beam path in air was 30 mm long, with the crystal in the center. Compared to the default setup, this arrangement has a significant shorter air beam path and the background caused by air-scattering is reduced. The sample-to-detector distance was set to 100 mm. For further background correction, 46 frames were collected under the same conditions without the sample. These frames were averaged and used as background in further processing. The data collection was run as a 180° ω-scan using 0.2° rotation and 2 min exposure times, resulting in 900 measured frames. Reference frames were collected every 2 h using 1 min exposures over a 10° ω rotation. Evaluation of the 29 reference frames did not show any significant change of the intensities.

The experimentally determined background was subtracted from all measured raw data frames. Furthermore, a masking procedure was applied to flag overexposed spots. *XDS* (Kabsch, 2010 ▸) was used to determine the orientation matrix for further processing with a modified version of *Xcavate* (Estermann & Steurer, 1998 ▸; Estermann, 2001 ▸). Intensity scaling of the original 32-bit images was obtained with *Xcavate*, and shading of non-measured areas and extraction of line profiles were performed with *ImageJ* (Abràmoff *et al.*, 2004 ▸). One-dimensional streak profiles were extracted by manually determining the lateral center of the streaks and summing over 20 pixels segments perpendicular to the streaks.

Further diffraction experiments on a crystal (70 µm × 70 µm × 80 µm) kept for six years in gelatin at room conditions have been performed at the X06DA beamline of the Swiss Light Source (Paul Scherrer Institute, Villigen, Switzerland). Monochromated radiation of 0.7085 Å was utilized to collect 1800 data frames during a 180° rotation of the crystal (0.3 seconds per frame) using a Pilatus 2M-F detector. Data collection was controlled by *DA+* (Wojdyla *et al.*, 2018 ▸), evaluation of the orientation matrix and reconstruction of the reciprocal space layers were performed using *XDS* and *Xcavate*.

Details of the data collections are summarized in Table 1 ▸.

### 2.3. Refinement   

The crystal structure of Mg(H_2_O)_2_[TeO_2_(OH)_4_] was solved using the charge flipping method implemented in *SUPERFLIP* (Palatinus & Chapuis, 2007 ▸) and refined against *F* ^2^ in *Jana2006* (Petříček *et al.*, 2014 ▸). Owing to disorder, the H atoms could not be located reliably and thus were not considered in the refinements. All atoms were refined using anisotropic atomic displacement parameters (ADPs). More details on different modeling and refinement attempts are given below (§3.8).

### 2.4. Calculation of diffuse scattering   

Experimental peak broadening of the one-dimensional intensity profiles was estimated by fitting Gaussian distributions to sharp reflections using the least squares (LS) solver *Ceres* (Agarwal *et al.*, 2020 ▸) refining the origin, reciprocal basis vector length, variance σ (all in pixels) and the individual intensities (in arbitrary units). The overall peak shape of the sharp reflections was well described by a Gaussian, only the base was better described by a Lorentz (Cauchy) distribution. One-dimensional diffuse scattering was calculated using the analytical expressions derived below. Atomic coordinates and ADPs of single layers were taken from the single-crystal refinements. The atomic form factors were calculated using polynomial approximations tabulated in *International Tables for Crystallography* (Brown *et al.*, 2006 ▸). Calculations were performed on a one-dimensional grid with four times the resolution of experimental data and later downsampled to the experimental grid.

Correlation parameters were estimated using a simple coordinate-descent algorithm optimizing in turn the origin (in pixels), the length of the reciprocal basis vector (in pixels) and the correlation parameter (unitless). Each variable was determined using a golden-section search. When multiple rods were refined concurrently, a hierarchical coordinate-descent was performed. In an outer loop, the correlation parameter was refined, in an inner loop the origin and basis vector length of each rod.

The validity of such a trivial search was confirmed by noting that the loss function possesses a single local minimum in each coordinate. The process was stopped when the change in all variables fell below a threshhold of 0.001 in the respective unit. The scale factor was determined after each cycle using a simple linear least-squares regression with unit weight, which also provided the loss function 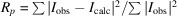 . Refinements using the weighting functions *w* = 1/(*I*_obs_)^*e*^ (*e* = 1,2), which are used in powder diffraction (Toraya, 1998 ▸), led to unreasonable peak shapes owing to an exaggerated emphasis on the intensities of ‘valleys’ (local minima between peaks).

### 2.5. Thermal analysis   

Simultaneous thermal analysis (STA) measurements in the temperature range 30–900°C were performed with a ∼50 mg sample in a corundum crucible on a NETZSCH STA 449 C Jupiter system coupled with a Aeolos quadrupole mass analyzer. The quartz capillary was kept at 250°C. The measured mass signals were 2 (H_2_), 12 (C), 14 (N), 15 (CH_3_), 16 (CH_4_, O), 17 (OH), 18 (H_2_O), 28 (N_2_, CO), 32 (O_2_) and 44 (CO_2_). All measurements were performed under a flowing argon atmosphere (20 ml min^−1^) and heating rates of 10 K min^−1^. Base line corrections of the TG curves were carried out by measuring the empty alumina crucible prior to each measurement. Temperature-dependent powder X-ray diffraction measurements (PXRD) were performed on a PANalytical X’Pert PRO diffractometer using a HTK1200 Anton-Paar high-temperature oven chamber mounted on the diffractometer. Prior to the measurement, the sample was finely ground and placed on a glass ceramic (Marcor) sample holder (depth 0.5 mm). The zero point was calibrated with a LaB_6_ standard and automatically adjusted during the measurements with a PC-controllable alignment stage. The samples were heated under atmospheric conditions at 10 K min^−1^ to the respective measurement temperature and kept for 5 min before measurement of each step to ensure temperature stability.

## 3. Results and discussion   

### 3.1. Crystal chemistry   

**Figure 1:**
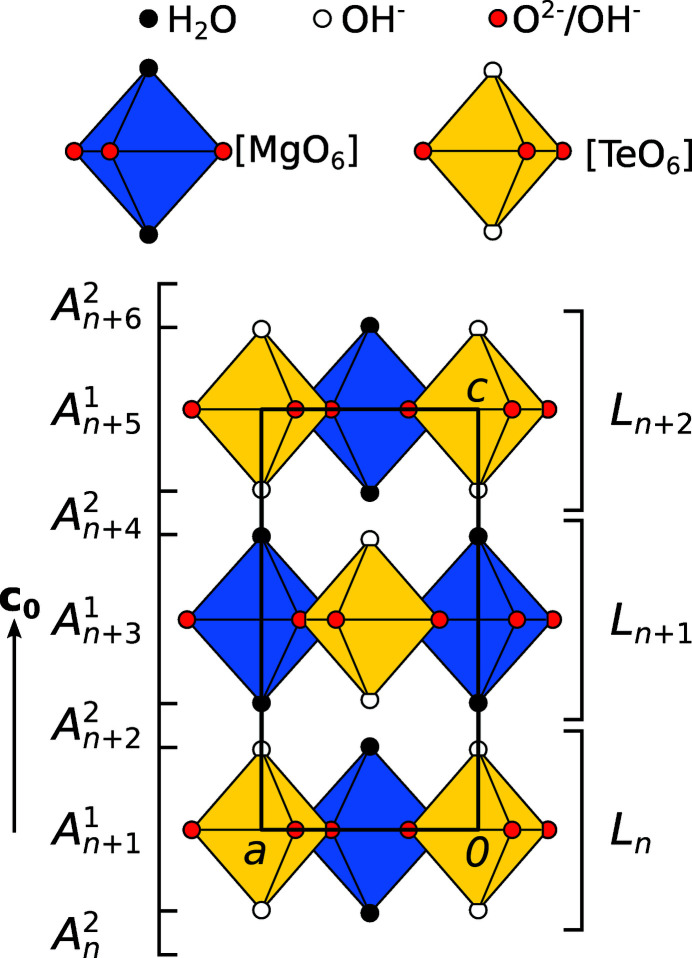
A polytype of Mg(H_2_O)_2_[TeO_2_(OH)_4_] [MDO_2_ (see §3.4), *Pcnm*, **c** = 2**c**_0_] viewed down [010]. Layer names according to the crystallo-chemical and the OD description are indicated to the right and left, respectively.

**Figure 2:**
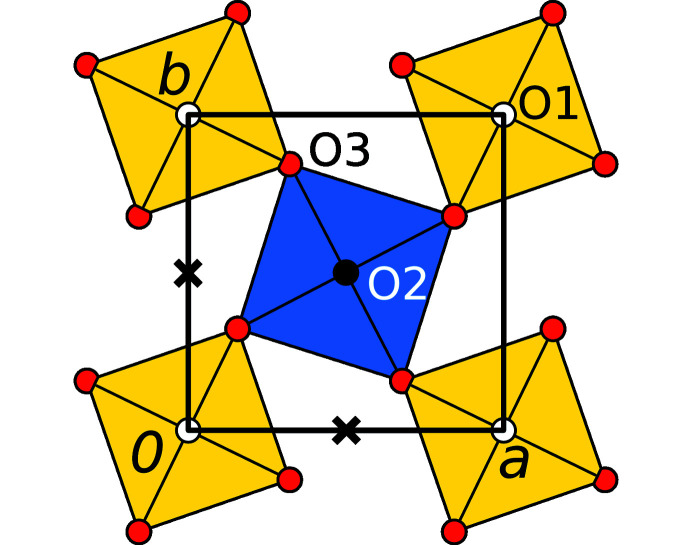
Idealized *L*_*n*_ layer in Mg(H_2_O)_2_[TeO_2_(OH)_4_] with *p*4/*m* symmetry viewed down [001]. Color codes as in Fig. 1 ▸. Crosses indicate the possible origins of the adjacent layers up to layer translation.

Mg(H_2_O)_2_[TeO_2_(OH)_4_] crystallizes as polytypes composed of distinct crystallo-chemical layers, designated as *L*_*n*_, where *n* is a sequential number (Fig. 1 ▸). The *L*_*n*_ layers possess (idealized) *p*4/*m* symmetry (Kopsky & Litvin, 2006 ▸) with a square lattice spanned by (**a**, **b**). **c**_0_ is the vector perpendicular to the layer planes with the length of one layer width. Henceforth, all directions and Miller indices will be given with respect to the basis (**a**, **b**, **c**_0_). The *L*_*n*_ layers are composed of close to regular [*M*O_6_] (*M* = Mg, Te) octahedra, which are connected by corners forming sheets (Fig. 2 ▸).

Both octahedra are located on sites with symmetry 4/*m* and are tilted by ∼26° in opposite directions about [001], thus leaving rhomboidal voids with a long and a short diameter of ≃5.0 and ≃2.5 Å, respectively. The *M* positions are alternately occupied with Te and Mg atoms in a checkerboard pattern. The O atoms connected only to Te and Mg are labeled O1 and O2, respectively. The shared O atom is O3 (Fig. 2 ▸). The [MgO_6_] octahedron is slightly larger than the [TeO_6_] octahedron with an average Mg—O distance of 2.056 Å compared to the average Te—O distance of 1.929 Å. Selected distances and angles are compiled in Table 2 ▸.

The rigid conformation of the octahedral [TeO_6_]^6−^ anion and the Te—O distances are characteristic for oxotellurates(VI). Reviews on the crystal chemistry of these compounds were given by Kratotochvíl & Jenšovský (1986 ▸), Loub (1993 ▸), Levason (1997 ▸) and Christy *et al.* (2016 ▸). An octahedral coordination is the most common coordination for Mg^II^ cations, and the average Mg—O distance of 2.057 Å compares well to the maximum of the distribution of Mg—O distances of 2.1 Å given in a survey on Mg—O coordination polyhedra (Blatov *et al.*, 1999 ▸; Gagné & Hawthorne, 2016 ▸).

Bond valence sums (BVSs) are a useful tool to assign H atoms, in particular for those cases where H atoms cannot be located, *e.g.* in the presence of heavy atoms, from X-ray diffraction data (Donnay & Allmann, 1970 ▸). Neglecting the contributions of H atoms, in the ideal case, the O atoms of H_2_O molecules, OH^−^ ions and O^2−^ ions have total BVS of 0, 1 and 2 valence units (v.u.), respectively. Bond valence calculations based on the 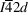 model of §3.8 with 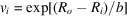 (Brown, 2002 ▸) using the parameters *R*_*o*_ = 1.693 Å, *b* = 0.37 for Mg—O and *R*_*o*_ = 1.917 Å, *b* = 0.37 for Te—O (Brese & O’Keeffe, 1991 ▸) result in BVSs of 0.84 v.u. (O1), 0.36 v.u. (O2) and 1.34 v.u. (O3). It has to be noted that these BVS calculations are slightly skewed by substitutional disorder of the Te and Mg atoms as well as positional disorder of the O atoms, showed by enlarged ADPs.

According to these BVSs, the Te atoms are bonded to two OH^−^ anions (O1) and the Mg atoms to two H_2_O molecules (O2). The remaining two H atoms per formula unit are connected to two out of four bridging O3 atoms, amounting to one per rhomboidal void. Thus, the structural arrangement of the compound can be expressed with the connectivity formula 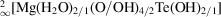 . This is in agreement with crystallo-chemical considerations and corresponds to an electronically neutral structure. Moreover, the Te—O bond lengths distribution in the [TeO_2_(OH)_4_]^2−^ octahedron is in good agreement with those of other structures comprising this type of anion (Weil, 2004 ▸, 2007 ▸; Weil *et al.*, 2017 ▸).

The larger and smaller than ideal BVSs of the H_2_O molecules and OH^−^ anions (0.36 and 0.83 versus 0 and 1 v.u.) can be explained by the H atoms being involved in hydrogen-bonding. Indeed, the distances between close O atoms [O1⋯O1 2.9316 (14) Å; O1⋯O2 2.9024 (14) Å; O2⋯O2 2.8746 (14) Å; O3⋯O3 2.525 (3) Å] strongly suggest formation of intra- and interlayer O—H⋯O hydrogen bonds.

### 3.2. Polytypism   

**Figure 3:**
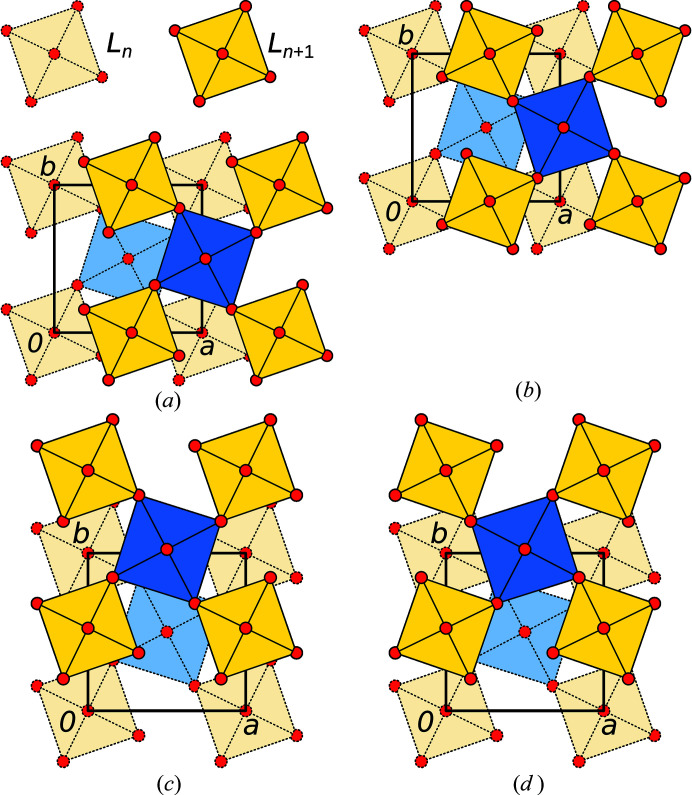
The four kinds of (*L*_*n*_,*L*_*n*+1_) layer pairs in Mg(H_2_O)_2_[TeO_2_(OH)_4_], viewed down [001]. The *L*_*n*_ layers are marked by brighter colors and dotted lines. In (*c*) and (*d*) the Te and Mg atoms in *L*_*n*+1_ are exchanged with respect to (*a*) and (*b*). In (*d*) and (*c*) the *L*_*n*_ and *L*_*n*+1_ layers do not, in (*b*) and (*d*) they do feature orientation inversion. Note that under the idealization of equal Mg—O and Te—O distances, the oxygen substructures are identical in the (*a*) and (*d*) as well as the (*b*) and (*c*) layer pairs.

The origin of the *L*_*n*+1_ layer is related to the origin of the adjacent *L*_*n*_ layer by a translation of 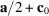 or 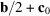 , as indicated in Fig. 2 ▸. In these two different stacking possibilities the locations of the Te and Mg atoms are exchanged. An alternation of the two will henceforth be called Te/Mg exchange. Moreover, every *L*_*n*_ layer can appear in two orientations related by *m*_〈100〉_ operations. A change in orientation will be called orientation inversion. The four resulting stacking possibilities are shown in Fig. 3 ▸.

### 3.3. Order–disorder description   

The order–disorder (OD) theory (Dornberger-Schiff & Grell-Niemann, 1961 ▸) has been devised to explain the common occurrence of polytypism in all classes of compounds. It is based on layers, which do not necessarily correspond to layers in the crystallo-chemical sense (Grell, 1984 ▸). The crucial point in an OD description is that pairs of adjacent layers are equivalent, which corresponds to the vicinity condition (VC). However, pairs of adjacent layers without [Figs. 3 ▸(*a*) and 3 ▸(*c*)] and with orientation inversion [Figs. 3 ▸(*b*) and 3 ▸(*d*)] are not equivalent and therefore violate the VC. The particular layer choice as described here is therefore not of the OD type.

An OD description can nevertheless be achieved by ‘slicing’ the structure into two kinds of layers, designated as *A* ^1^ and *A* ^2^ (Fig. 1 ▸, left). The structure then belongs to a tetragonal category IV OD family built of two kinds of non-polar (with respect to the stacking direction) layers.

The OD groupoid family symbol reads as[Chem scheme1] 
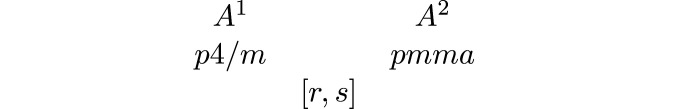


according to the notation of Grell & Dornberger-Schiff (1982 ▸).

The first line of the symbol gives the name of the layers, the second their symmetry and the third one possible arrangement of adjacent layers. Note that in OD theory, layer group symbols with five directions are sometimes necessary to describe tetragonal OD groupoid families. Here, because it is not necessary to distinguish between the [100] and [010] directions, as well as the [110] and 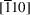 directions, the usual symbols can be used.

**Figure 4:**
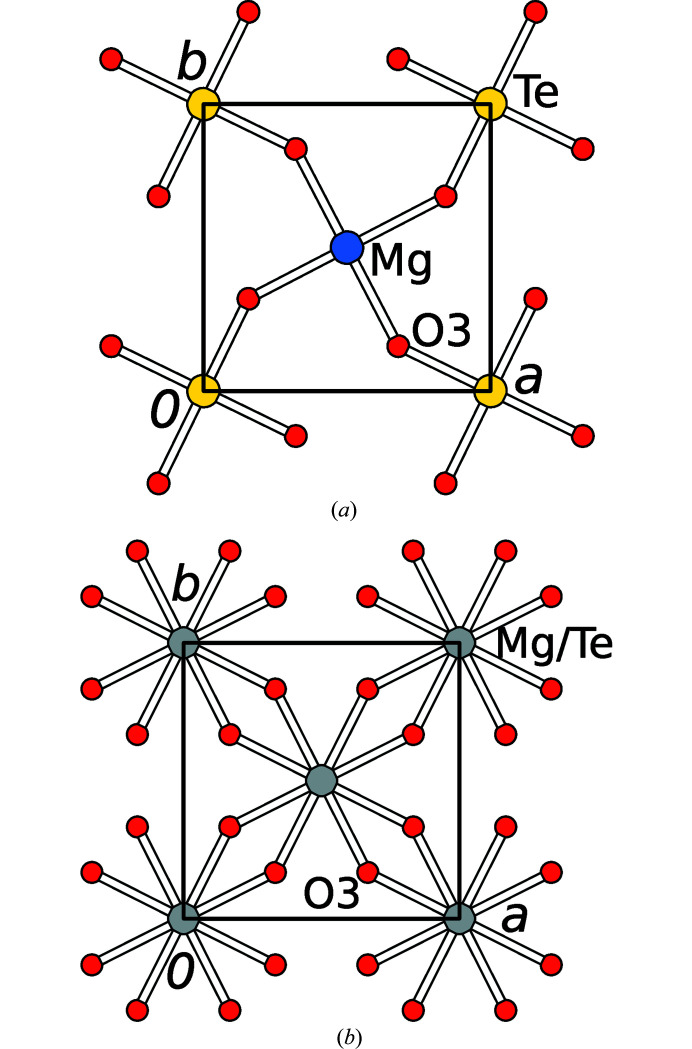
(*a*) OD layer *A* ^1^ of Mg(H_2_O)_2_[TeO_2_(OH)_4_] and (*b*) superposition of four *A* ^1^ layers in the family structure. Te, Mg and O atoms are represented by yellow, blue and red spheres of arbitrary radius. The O1 and O2 atoms above Te and Mg (with respect to [001]) are omitted for clarity. Disordered 1:1 Te:Mg positions are represented by gray spheres. In (*b*) the coordinates of the O3 atom have been idealized to fulfill the equation *x* + *y* = ½.

**Figure 5:**
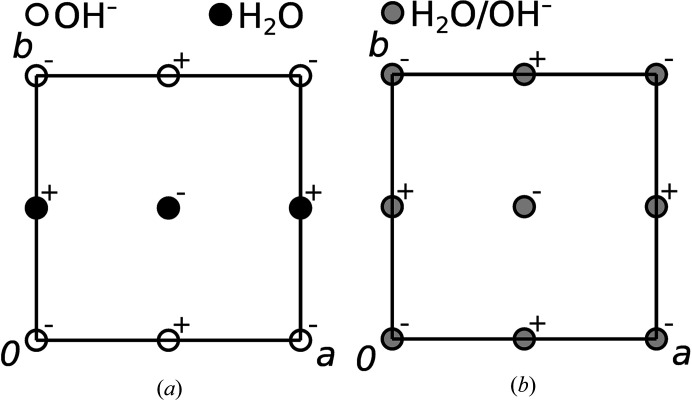
(*a*) OD layer *A* ^2^ of Mg(H_2_O)_2_[TeO_2_(OH)_4_] and (*b*) superposition of four *A* ^2^ layers in the family structure. OH^−^ anions and H_2_O molecules are represented by black and white spheres of arbitrary radius. A gray sphere is an equal superposition (assuming equal Te—O and Mg—O distances), where + and − symbols mark groups located above and below the drawing plane, respectively.

The *A* ^1^ layers possess *p*4/*m* symmetry. They are built of the [MgO_6_] and [TeO_6_] octahedra [Fig. 4 ▸(*a*)] and the disordered hydrogen atom belonging to O^2−^/OH^−^ in the rhombohedral void. The *A* ^2^ layers are built of the OH^−^ anions (O1) and H_2_O molecules (O2) that connect the *L*_*n*_ layers [Fig. 5 ▸(*a*)]. Thus, the O1 and O2 atoms are located at the layer interfaces and belong to both OD layers. The layer symmetries were deduced under the assumption of a disordered hydrogen-bonding network.

The third line of the symbol indicates that, in one possible arrangement, the origins of the 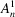 and 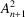 layers are spaced by *r* **a** + *s* **b** + **c**_0_/2. According to the stacking rules described above, (*r*, *s*) adopt the values 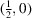 or equivalently 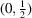 , *i.e.* the 4_[001]_ and 2_[001]_ axes of the *A* ^1^ and *A* ^2^ layers coincide. Note that in contrast to many other OD families, here the parameters adopt a precise value because adjacent layers share common atoms (O1 and O2) located on special positions.

The *NFZ* relationship (Ďurovič, 1997 ▸) is a formalism to determine the alternative stacking possibilities in a family of OD structures. It is based on the groups 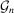 of those operations of the *A*_*n*_ layers that do not reverse the orientation with respect to the stacking direction (λ-τ-POs according to the OD terminology). For the *A* ^1^ and *A* ^2^ layers, 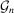 is *p*4 and *pmm*2, respectively. Because the adjacent layers are not equivalent, the *NFZ* relationship reads as 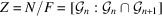 , where 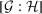 designates the index of the subgroup 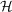 of 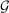 . For any pair of adjacent layers, 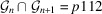 .

For an 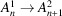 contact, *Z* = *N*/*F* = [*p*4 : *p*112] = 2. Thus, given an 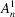 layer, the adjacent 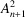 layer can appear in two orientations (with *pmma* and *pmmb* symmetry), which are related by the fourfold rotation of the 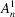 layer. For an 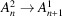 contact, *Z* = *N*/*F* = [*pmm*2 : *p*112] = 2. Given an 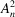 layer, the adjacent 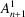 layer can likewise appear in two positions, which in this case are related by the *m*_〈100〉_ reflections of the 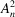 layer.

By following these stacking rules, an infinity of polytypes can be constructed, which are equivalent to the non-OD polytypes described in the previous section. The usefulness of the OD description does not only lie in the concise symmetry classification. It also sheds light on the crystallo-chemical reasons of the polytypism by splitting them into two distinct contributions. On the one hand ( 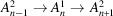 ), the orientations of the hydrogen-bonding network to both sides of the [TeO_6_] and [MgO_6_] octahedra may be the same, or different. On the other hand ( 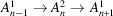 ), the inter-layer hydrogen bonding independent of the orientation of the octahedral sheets.

### 3.4. Maximum degree of order polytypes   

Polytypes of a maximum degree of order (MDO) are a central concept of OD theory (Dornberger-Schiff, 1982 ▸; Dornberger-Schiff & Grell, 1982 ▸). MDO polytypes cannot be decomposed into simpler polytypes, *i.e.* into polytypes composed only of a selection of pairs, triples or any *n*-tuples of adjacent layers. Experience shows that the majority of macroscopically ordered polytypes are of the MDO type.

There are two kinds of 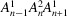 triples, namely with and without orientation inversion. Moreover, there are two kinds of 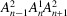 triples, namely with and without Te/Mg exchange.

The combination of these triples results in four MDO polytypes:

MDO_1_: never orientation inversion, never Te/Mg exchange, *B*112/*m*, **c** = 2**c**_0_;

MDO_2_: always orientation inversion, never Te/Mg exchange, *Pcnm*, **c** = 2**c**_0_;

MDO_3_: never orientation inversion, always Te/Mg exchange, *I*4_1_/*a*, **c** = 4**c**_0_;

MDO_4_: always orientation inversion, always Te/Mg exchange, 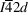 , **c** = 4**c**_0_.

All other stacking arrangements can be divided into fragments of MDO polytypes, which therefore represent the ‘alphabet’ of an OD family.

Atomic coordinates for all four MDO polytypes are listed in Table 3 ▸.

### 3.5. Family structure   

The family structure of an OD family is a fictitious structure in which all stacking possibilities are realized to the same degree. It plays an important role in the elucidation of OD structures. The family structure of Mg(H_2_O)_2_[TeO_2_(OH)_4_] has *F*4/*mmm* symmetry (non-standard setting of *I*4/*mmm*) with **c** = 2**c**_0_ (coordinates in Table 3 ▸).

For a fixed 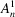 layer, the adjacent 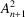 layer can appear in two orientations related by the 4_[001]_ operation. Each of these two orientations gives rise to two orientations of the 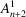 layer, which are related by the *m*_〈100〉_ operations of the 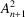 layer. Thus, in the family structure the *A* ^1^ layers are an equal superposition of four positions (Te/Mg disorder and orientation disorder) with *c*4/*mmm* (non-standard setting of *p*4/*mmm*) symmetry [Fig. 4 ▸(*b*)].

According to analogous reasoning in the *A* ^2^ layers of the family structure, the OH^−^ anions and H_2_O molecules are disordered in a 1:1 ratio [Fig. 5 ▸(*b*)]. These disordered layers possess *c*4/*emm* (non-standard setting of *p*4/*nmm*) symmetry.

### 3.6. Diffraction pattern   

**Figure 6:**
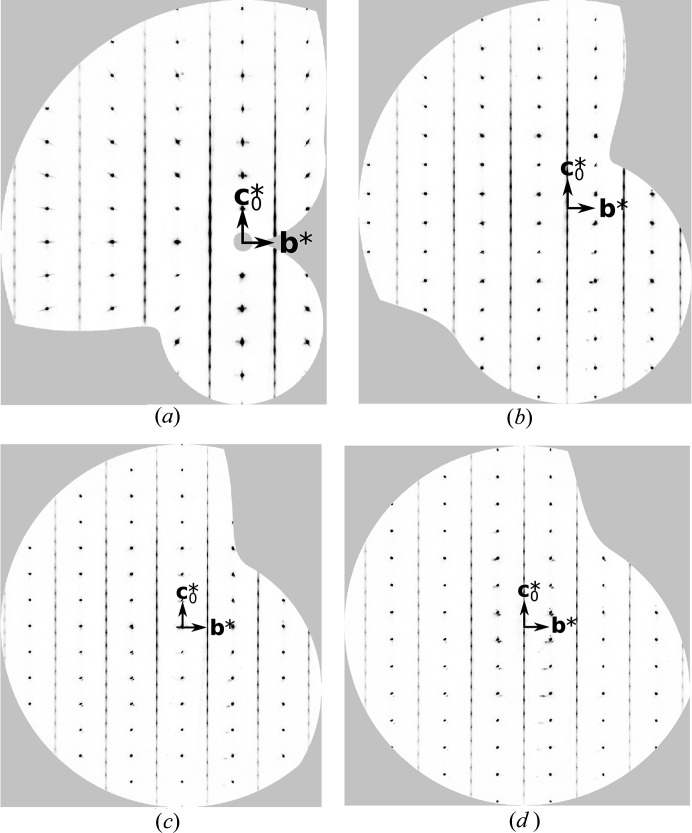
The *h* = 0…3 planes of reciprocal space of Mg(H_2_O)_2_[TeO_2_(OH)_4_] reconstructed from image-plate data. (*a*) *h* = 0, (*b*) *h*=1, (*c*)*h* =2, (*d*) *h* = 3.

The diffraction pattern of Mg(H_2_O)_2_[TeO_2_(OH)_4_] features rods with sharp reflections and rods with broader reflections on top of prominent one-dimensional diffuse scattering (Fig. 6 ▸). Such diffraction patterns are characteristic for polytypes with translationally equivalent layers (Jeffery, 1953 ▸; Ferraris *et al.*, 2008 ▸), and were the inspiration for the name ‘OD’ (Bragg reflections: order; streaks: disorder).

In Mg(H_2_O)_2_[TeO_2_(OH)_4_], the *L*_*n*_ layers are *not* translationally equivalent, since they can appear in two orientations. As will be shown below, in this case the reason of the rods lacking diffuse scattering lies in the particular makeup of the *L*_*n*_ layers, namely the similar size of the [MgO_6_] and the [TeO_6_] octahedra.

In the reciprocal basis 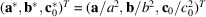 , the structure factor *F*(*hk*ν) of a polytype can be calculated as the sum of the structure factors *F*_*n*_(*hk*ν) of the individual *L*_*n*_ layers: 

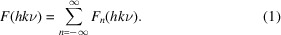

Since the translation lattices of all layers are spanned by (**a**, **b**), *F*_*n*_(*hk*ν) is only non-zero for 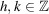 . The structure factor *F*_*n*_(*hk*ν) can be decomposed into the contributions 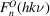 of the O3 atom and 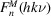 of the remaining atoms (Te, Mg, O1, O2): 

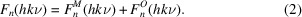

The origin of the *L*_*n*_ layer can be written as 

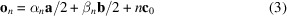

with 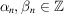 and α_0_ = β_0_ = 0. Since the origin shift from *L*_*n*_ to *L*_*n*+1_ is either **a**/2 + **c**_0_ or **b**/2 + **c**_0_, α_*n*_ + β_*n*_ is even, if and only if, *n* is even, which can be expressed by 

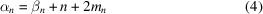

with 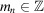 .

The Mg, Te, O1 and O2 atoms are not affected by orientation inversion, since the eigensymmetry of their (layer group) orbits is *p*4/*mmm*, which contains the reflection relating both orientations. These parts of the layers are therefore obtained from the *L*_0_ layer by translation along **o**_*n*_. According to equations (3) and (4), 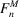 can therefore be written in terms of 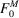 as 

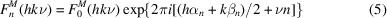




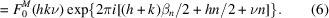

Note that since Mg, Te, O1 and O2 are located on fourfold rotation axes, their displacements are isotropic in the (001) plane and, therefore, disregarding desymmetrization, the reflection at [100] has no influence on their (harmonic) ADPs.

The orientation of the *L*_*n*_ layer will be described by ω_*n*_ = 0, 1, 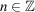 . If O3 is located on the 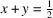 line, which is perfectly realized if *d*(Te—O3) = *d*(Mg—O3), ω_*n*_ = 1 corresponds to an additional translation of (**a** + **b**)/2 with respect to **o**_*n*_. If the displacement of the O3 atom is likewise isotropic in the (001) plane, 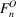 can be written in terms of 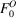 as 

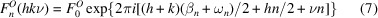

If *h* + *k* is even, then (*h* + *k*)β_*n*_ and (*h* + *k*)(β_*n*_ + ω_*n*_) are likewise even and equations (5) and (7) simplify to 

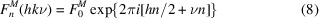

and 

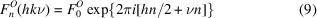

and therefore 

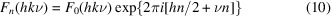

and 

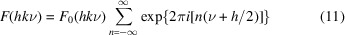




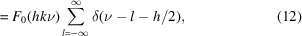

where δ is the Dirac delta distribution. Note that the last equals sign represents an abuse of notation as the given function series does not converge at any point [technically, the series converges in the distributional sense (Bricogne, 2010 ▸)].

In summary, on rods *h* + *k* even only sharp reflections are observed at ν = *l*/2, 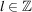 , where *h*, *k* and *l* are all even or all odd. This corresponds to the diffraction pattern of a crystal with a tetragonal *F*-centered (*tF*) lattice with the centered reciprocal basis 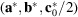 . These reflections correspond to the diffraction pattern of the family structure (§3.5) and are called the family reflections. All stacking arrangements, ordered or disordered, contribute equally (proportional to their volume fraction) to these reflections, since neither α_*n*_, β_*n*_ nor ω_*n*_ contribute to equation (12).

On rods *h* + *k* odd the simplifications above do not apply and diffraction intensities can appear at arbitrary positions. Bragg reflections on these rods are called characteristic reflections, because they are generated only by certain polytypes. The characteristic reflections of MDO_1/2_ are located at ν = *l*/2, 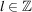 , those of MDO_3/4_ at 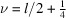 , 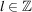 .

**Figure 7:**
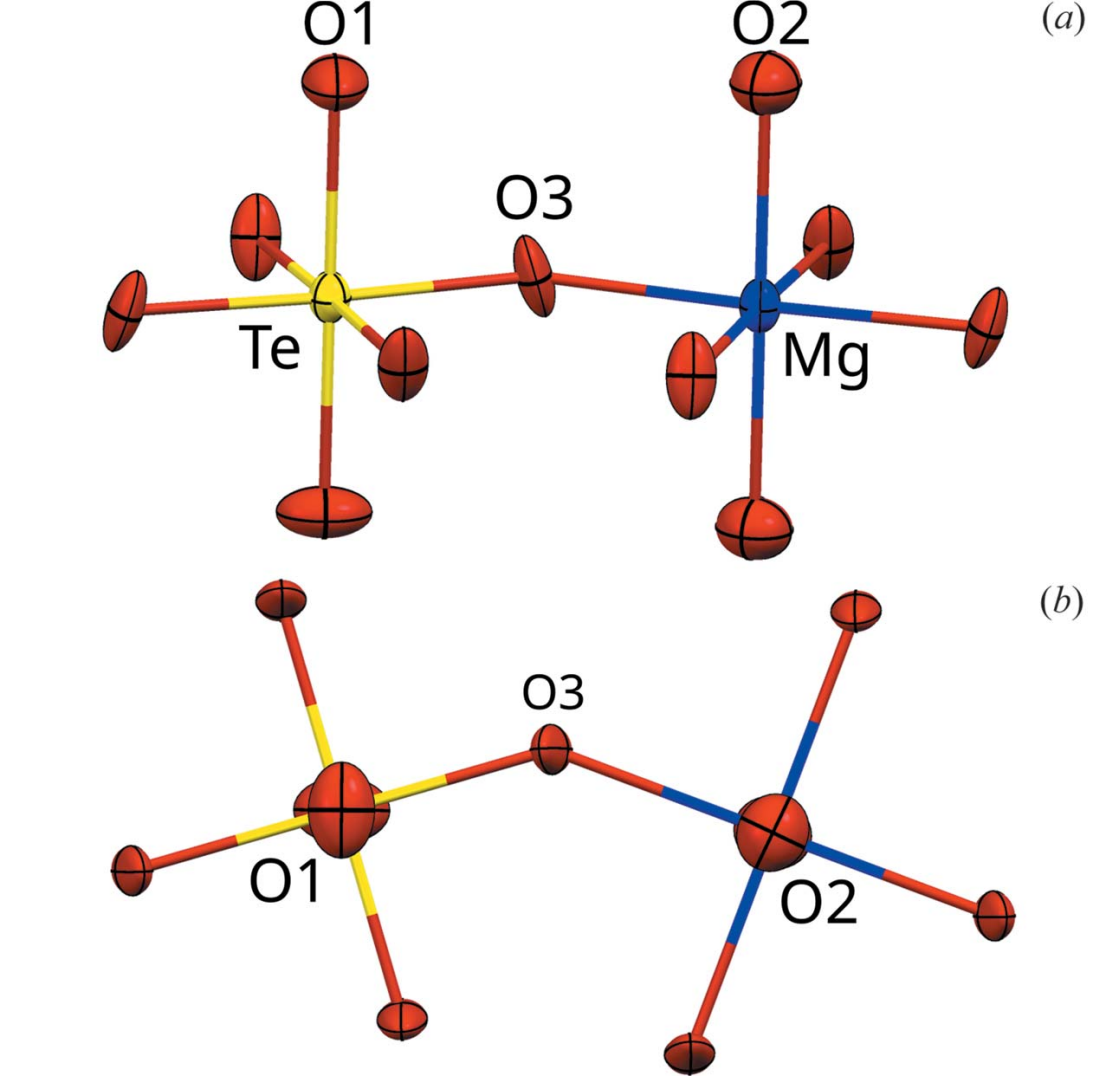
Fragment of the Mg(H_2_O)_2_[TeO_2_(OH)_4_] structure, showing the ADPs as ellipsoids drawn at the 75% probability level (Te: yellow, Mg: blue, O: red). Data taken from the 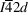 refinement.

The calculations above were derived under the assumption that *d*(Te—O3) = *d*(Mg—O3), whereas the actual structure deviates from this assumption [1.906 (3) versus 2.064 (2) Å]. Moreover, ordered polytypes typically feature desymmetrization (Ďurovič, 1979 ▸). For example, in the MDO_3/4_ polytypes, the O3 atom is located slightly off the *z* = 0 reflection plane of the *p*4/*m* layer symmetry (Table 3 ▸). Distinctly enlarged ADPs of the O1 and O2 atoms show that they are located on the fourfold axis (which is a twofold axis in the actual MDO_3/4_ polytypes) only on average (Fig. 7 ▸). These deviations may lead to violations of the systematic non-crystallographic absences, namely faint streaking and very weak characteristic reflections on *h* + *k* even rods.

### 3.7. Rods with insignificant contribution of O3   

To differentiate between the effects of orientation inversion and Te/Mg exchange on the diffraction pattern, it is useful to note that the fractional coordinates *x* and *y* of the O3 atom, which is essentially the only atom affected by orientation inversion, are close to 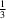 and 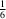 , respectively. If the O3 atom is idealized as being located on such a position, the *L*_0_ layer contains up to translation two O3 atoms at 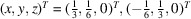 and the two atoms obtained by inversion at the origin. If, moreover, the displacements of the O3 atom are considered as being isotropic in the (001) plane [ 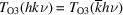 ], then the structure factor 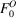 is 

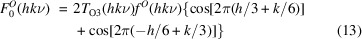

where *f* ^*O*^(*hk*ν) is the atomic form factor of O. Note that the structure factor is real and contains only 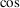 terms owing to the inversion at the origin. If *h* is divisible by three, *i.e.* *h* = 3*h*′, 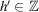 , this expression simplifies for rods *h* + *k* odd to 

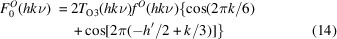




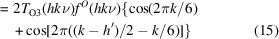




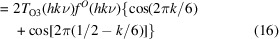




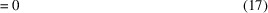

(*h*′ is even, if and only if, *h* is even and therefore *k* − *h*′ is odd). The same argument can be applied to rods with *k* divisible by three. Thus for *h* or *k* divisible by three, equation (2) simplifies to 

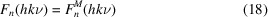

and equation (1) can ultimately be written as 

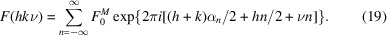

The variable ω_*n*_, which describes the orientation of the octahedra, does not affect rods *h* + *k* odd with either *h* or *k* divisible by three, and any significant diffuse scattering or Bragg reflections on these rods are due to the arrangement of the Te and Mg atoms.

**Figure 8:**
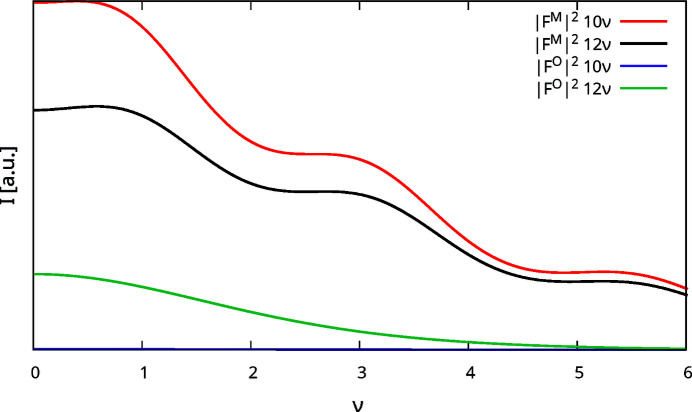
Plots of 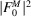 and 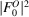 against ν for the 10ν and 12ν rods. Only ν ≥ 0 are shown, because the structure factors are essentially symmetric by reflection at the ν = 0 plane. 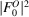 is practically 0 and therefore barely to be seen at the bottom of the chart.

To illustrate the effect in absolute terms, plots of 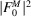 and 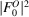 against ν are given in Fig. 8 ▸ for the 10ν and 12ν rods. Indeed, for 10ν the contribution of 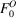 is negligible when compared to 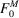 . On the other hand, 12ν features significant contribution at low scattering angles. In particular, this rod (including symmetry equivalents) has the highest relative contribution of 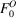 .

### 3.8. Classical refinements   

To determine atomic coordinates and ADPs, classical independent atom model refinements were performed. In a first refinement, only the family reflections were considered. Excellent reliability factors are thus obtained (Table 4 ▸). In the family structure, Te/Mg exchange and orientation inversion are realized in 50% of the layers. In principle, the O3 atom is disordered over four positions. Nevertheless, only two positions could be resolved, because the Te—O3 and Mg—O3 distances are nearly equal. Likewise, the O1 and O2 atoms could not be separated without introduction of distance restraints and therefore were refined as a single O1/O2 position.

On top of the lines of diffuse scattering at *h* + *k* odd are located elongated peaks where the characteristic reflections of MDO_3/4_ are expected (Fig. 6 ▸). No peaks corresponding to MDO_1/2_ were observed (though see §3.13). One has to realize that treating these peaks as Bragg reflections in classical refinements will inevitably introduce systematic errors.

Owing to the systematic non-space group absences (§3.6), reflection conditions cannot differentiate between MDO_3_ (*I*4_1_/*a*) and MDO_4_ ( 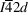 ). Moreover, owing to diffuse scattering on rods *h* + *k* odd, intensities in violation of the *I*-centring are observed. Thus, classical space group determination is unreliable and the diffractometer software strongly suggests the space group *I*4_1_/*amd* (**c** = 4**c**_0_), which is the space group of a 1:1 superposition of MDO_3_ and MDO_4_.

The first model was generated and refined using this *I*4_1_/*amd* symmetry, where the O3 atom is disordered with a 1:1 occupation ratio about the *m*_[100]_ reflection plane. To achieve satisfying residuals, occupational disorder of the Te and Mg atoms (with Mg′ and Te′) had to be introduced, corresponding to a contribution of MDO_1/2_ fragments.

Based on the refined model in *I*4_1_/*amd*, the symmetry was reduced by an index of 2 to *I*4_1_/*a* (MDO_3_) and 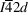 (MDO_4_), respectively. The linear parts of the lost operations were retained as the twin law and the twin volume ratio was refined. The disordered O3 position was split in both cases into two distinct positions (O3 and O3′). The coordinates and ADPs of O3 and O3′ were constrained to be equal with respect to the *m*_[100]_ operation and the occupancies were refined and constrained to a sum of 1. A comparison of the refinements is given in Table 4 ▸. The volume fraction of MDO_3/4_ (as opposed to MDO_1/2_) was derived from the occupancy of Te as |2occ(Te) − 1|. Likewise, the volume fraction of the major domain of the MDO_3_/MDO_4_ pair was derived as |2occ(O3) − 1| (see Appendix *B* [App appb]).

According to these refinements, there was ∼10–15% of MDO_1/2_ present in the crystal under investigation. Estimating the MDO_3_:MDO_4_ ratio is more difficult. According to the refinement with the best reliability factors ( 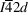 ), there are approximately equal amounts of MDO_3_ and MDO_4_, which would correspond to a 50% chance of orientation inversion. The *I*4_1_/*a* refinement on the other hand suggests an MDO_3_:MDO_4_ ratio of ∼2:1, which shows the difficulty of deriving these values from routine refinements. The fundamental problem is that a disordered stacking is in general not equivalent to a superposition of MDO polytypes.

Allotwinning, *i.e.* the association of macroscopic domains of distinct polytypes (Nespolo *et al.*, 1999 ▸), was ruled out owing to diffuse scattering. Indeed, such models did not lead to improved reliability factors. Likewise, placing the characteristic and family reflections on different scales to avoid the Ďurovič effect (Nespolo & Ferraris, 2001 ▸) led to unreliable refinements because the ratio of polytypes and the ratio of the scales correlate (Hans *et al.*, 2015 ▸). As will be shown below (§3.10) Te/Mg exchange does occur and thus the single-scale refinements are preferred, even though the quantification of Te/Mg exchange is inaccurate.

In summary, neither the amount of MDO_1/2_ nor the MDO_3_:MDO_4_ ratio can be quantified reliably with routine refinements, demonstrating the inherent difficulties of structurally characterizing such compounds. Nevertheless, these refinements are crucial to determine Mg—O and Te—O distances.

### 3.9. Disorder model   

To quantify the diffuse scattering, a simple growth model was derived from the OD interpretation given in §3.3. The crystal is described as an alternating succession of *A* ^1^ and *A* ^2^ OD layers. According to the OD description, pairs of adjacent OD layers are geometrically equivalent, but triples may differ. Therefore, in the simplest growth model the *A*_*n*_ layer depends on the *A*_*n*−1_ and *A*_*n*−2_ layers. Since there are two kinds of 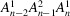 triples and two kinds of 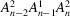 triples (§3.4), this model is fully determined by two parameters. *P*_MgTe_ describes the probability of 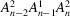 without Mg/Te-inversion and *P*_orient_ the probability of 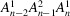 triples without orientation inversion. In some cases, it will be more convenient to express these probabilities in terms of the correlation coefficients

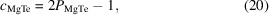




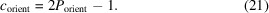

This two-parameter model is sufficient to describe all four MDO polytypes and also of equal overlays of MDO polytypes, as listed in Table 5 ▸.

In this trivial model, each layer triple is considered independent of the previous triple. In more refined models, the orientation–inversion probability could depend on the occurrence of Mg/Te inversion and vice versa. Additional parameters would then be required.

Growth models are conveniently expressed as Markov chains (Welberry, 2010 ▸). The above model corresponds to the four-state Markov chain 
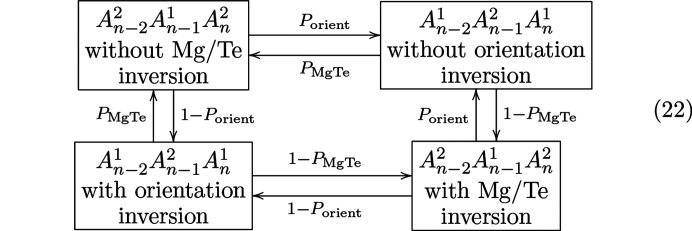


where each step corresponds to a new triple of OD layers, which has two OD layers in common with the previous triple. This Markov chain has a period of two since an *A* ^1^ layer is only added after every second step (and likewise for *A* ^2^). Such chains are developed into two (or more for higher periods) independent chains, here from the *n*th triple to the *n*+2nd triple. These two Markov chains are most conveniently expressed in terms of the crystal-chemical *L_n_* layers: 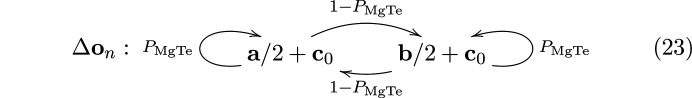 
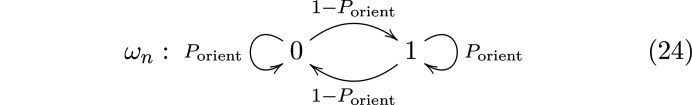


The first Markov chain describes the relation of the origin of the *L_n_* and *L*_*n*+1_ layers and the second chain the orientation of the *L_n_* layer. The chains are independent, because one OD layer triple does not depend on the previous triple. Each chain can be considered as an independent nearest-neighbor model, since the **o**_*n*_ depends only on **o**_*n*−1_ and ω_*n*_ on ω_*n*–1_.

For *P*_MgTe_, *P*_orient_ ≠ 0,1, the Markov chains converge to the equilibrium states *P*(Δ**o**_*n*_ = **a**/2) = *P*(Δ**o**_*n*_ = **b**/2) = *P*(ω_*n*_ = 0) = *P*(ω_*n*_ = 1) = ½, *i.e.* after an infinity of layers, both origin shifts and both layer orientations are equally likely. In the following only this general case will be considered.

### 3.10. Diffuse scattering   

To calculate the diffraction pattern of disordered structures, it is advantageous to directly calculate the intensity *I*(*hk*ν) = |*F*(*hk*ν)|^2^ in terms of pair correlations between layers (Welberry, 2010 ▸): 

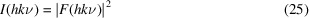




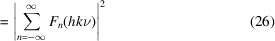




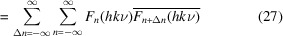

where an overline designates the complex conjugate. The orientation of the *L_n_* layer is flipped with respect to the *L*_0_ layer if ω_*n*_ = 1. To express the structure factor of such a layer, it will be related to the structure factor 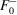 of the mirrored *L*_0_ layer:

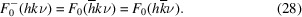

The *hk*ν argument of *F* will henceforth be omitted for brevity.

The diffraction intensity can then be expressed in terms of probabilities:

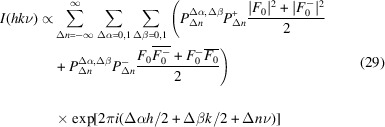




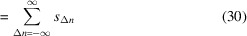

where 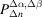 expresses the probability that the origins of the *L_n_* and *L*_*n*+Δ*n*_ layers are separated by Δα_*n*_ **a**/2 + Δβ_*n*_ **b**/2 + Δ*n* **c** (up to a full layer lattice translation). 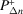 and 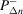 are the probabilities that the *L_n_* and *L*_*n*+Δ*n*_ layers possess the same, respectively opposite, orientation. 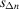 , the sum over Δα and Δβ, is the pair-correlation function of layers spaced by 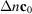 . It should be stressed that equation (29) is only valid for an equal probability of both layer orientations (*P*_orient_ ≠ 0, 1, large domain size) and independence of both Markov chains. On the flip side, it is valid for more complex growth models with interactions over more than one layer width.

From the stacking rules it follows that 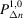 = 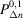 = 0 for Δ*n* even and 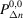 = 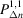 = 0 for Δ*n* odd. Since, as has been shown above, significant diffuse scattering is only observed on rods *h* + *k* odd, let us concentrate on these. Then, the exponential factor of the Δα = Δβ = 1 terms in equation (29) is 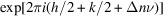 = 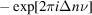 . Factoring out the probabilities, for Δ*n* even we thus obtain

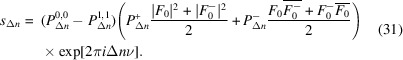

In analogy, for Δ*n* odd and *h* + *k* odd, 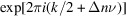 = 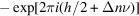 (if *k* is odd *h* is even and vice-versa) and therefore

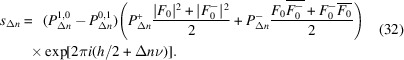

Let us now derive the ‘pair distribution’ probabilities. Obviously, the starting state of the growth model is 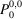 = 1 and 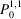 = 0. As has been noted above, in non-degenerate cases (*P*_MgTe_ ≠ 0,1), the equation (23) converges to an equilibrium state where the origin shifts Δ**o**_*n*_ = **a**/2+ **c**_0_ and Δ**o**_*n*_ = **b**/2 + **c**_0_ are equally likely and therefore 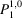 = 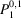 = ½. Repeated application of equation (23) to theses initial states gives the general case (see Appendix *C* [App appc]):

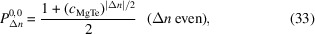




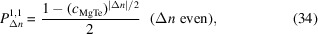




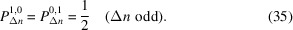

Note that for negative Δ*n*, the same reasoning applies and therefore the absolute value of Δ*n* is used in the exponents. In analogy, according to the Markov chain equation (24) the probabilities describing the orientations are

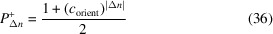




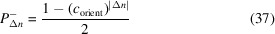




By substituting equation (35) into equation (32) it follows that *s*_Δ*n*_ = 0 for odd Δ*n*. Note that this is only valid for the simple nearest-neighbor model of equation (23). In more general growth models, these terms adopt non-zero values.

For even Δ*n*, from equations (33) and (34) it follows that 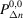 − 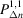 = (*c*_MgTe_)^|Δ*n*|/2^ and equation (31) becomes

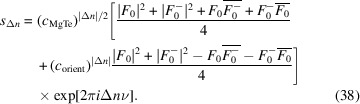




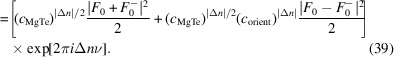

Ultimately, the intensity on rods *h* + *k* odd therefore is [see equation (30)]

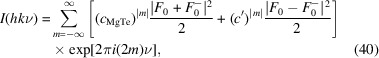

where *m* = Δ*n*/2 and

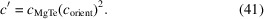

By identifying two geometric series (see Appendix *D* [App appd]), an analytical expression of *I*(*hk*ν) can be given as

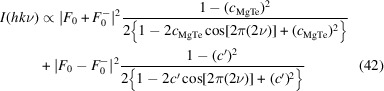

To avoid the unwieldy expressions in the parentheses, we will introduce the function family

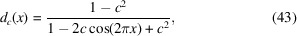

which describes the shape of one-dimensional diffuse scattering produced by a structure with a simple nearest-neighbor correlation of −1 < *c* < 1 (Welberry, 2010 ▸). *d_c_* (*x*) is generally (except for *c* = 0) a function with periodicity 1, featuring peaks which are sharper for increasing |*c*|. For |*c*| approaching 1, *d_c_* (*x*) converges to a Dirac comb with sharp reflections for integer *x* (*c* → 1) or half-integer *x* (*c* → −1). For *c* = 0, *d*_*c*_ is the constant function *d*_0_(*x*) = 1.

Using *d*_*c*_, equation (42) simplifies to

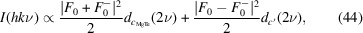

which shows that the diffuse scattering is the sum of two independent shape-functions of the nearest-neighbor correlation *c*_MgTe_ and *c*′. The factor 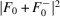 corresponds to the (hypothetical) intensity of an superposition of both orientations of the *L*_0_ layer and 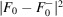 to the intensity of the difference of the electron density of these two orientations.

Since *c*′ depends on the square of *c*_orient_ [equation (41)], *c*_MgTe_ and *c*′ are of the same sign [sgn(*c*_MgTe_) = sgn(*c*′)]. Thus, the location of the peaks depends only on *c*_MgTe_, but not on *c*_orient_. For *c*_MgTe_ > 0, *I*(*hk*ν) has peaks at ν = *l*/2, 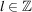 and for *c*_MgTe_ < 0 at ν = *l*/2 + 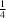 , 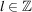 as is expected for MDO_1/2_-like and MDO_3/4_-like stacking arrangements, respectively.

Moreover, note that from |*c*_orient_| < 1 follows that |*c*′| < |*c*_MgTe_| and therefore ordering of the orientation inversion can never lead to sharper peaks for a given *c*_MgTe_, whereas its disorder can lead to more diffuse peaks.

But most remarkably, under the given assumptions (nearest-neighbor model, negligible desymmetrization, *c*_MgTe_, *c*_orient_ ≠ ± 1) the diffuse scattering is identical for pairs of *c*_orient_ with the same absolute value.

### 3.11. Estimation of the correlation coefficients   

Assuming the idealization *d*(Te—O3) = *d*(Mg—O3), orientation inversion corresponds to a translation of O3 by (**a** + **b**)/2 (see § 3.6). Using the decomposition 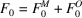 , 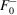 then is

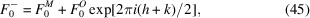

which for *h* + *k* odd becomes 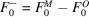 and consequently

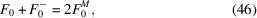




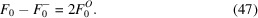

Thus, equation (44) becomes

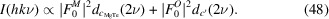

Conveniently, as has been shown above, 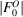 is negligible for rods *h* + *k* odd with *h* or *k* divisible by three (Fig. 8 ▸). Thus, these rods can be used to estimate *c*_MgTe_ with only a negligible contribution of *c*_orient_.

**Figure 9:**
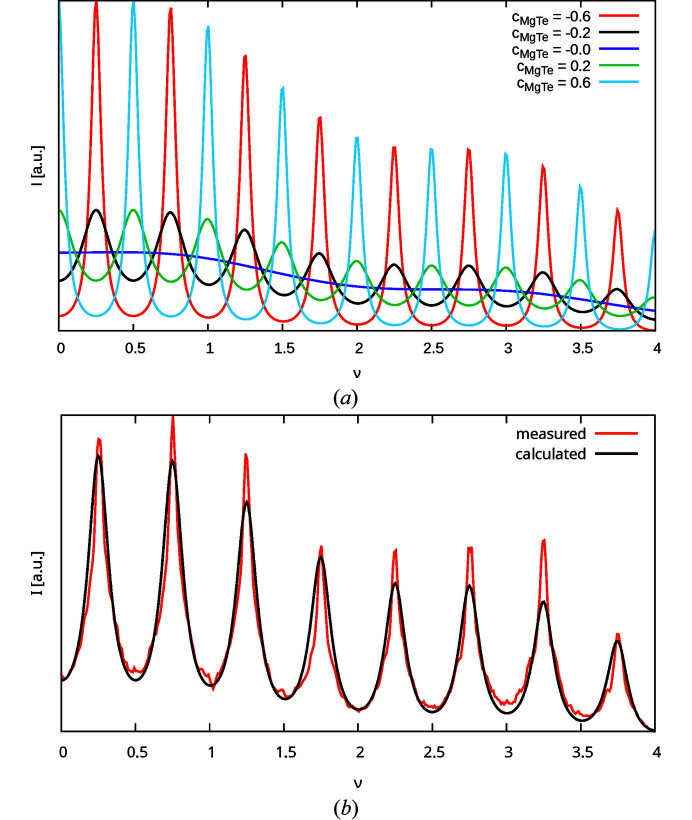
Intensity *I*(10ν) of the 10ν rod (*a*) calculated for various *c*_MgTe_ values and (*b*) with *c*_MgTe_ optimized against experimental data. Intensity is absolute, *i.e.* zero intensity is at the bottom of the chart.

Fig. 9(*a* ▸) gives *I*(10ν) plots for different values of *c*_MgTe_ calculated using only the 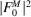 term of equation (48). To estimate *c*_MgTe_, a simultaneous LS optimization was performed on the rods listed in Table 6 ▸. Fig. 9 ▸(*b*) shows the result of the LS optimization without convolution of the experimental peak shape for the 01ν rod. As expected, an additional convolution with the experimental peak shape results in a slightly more negative correlation *c*_MgTe_ (−0.353 versus −0.338). Since the refinements without convolution result generally in better fits (Table 6 ▸), we will henceforth assume the latter value.

The agreement of the experimental and calculated curves is reasonable, though not perfect as the experimental peaks are somewhat narrower. Even though a stronger negative correlation *c*_MgTe_ leads to narrower peaks, it is in disagreement with the strong diffuse scattering between the peaks. We suppose that the sample is composed of domains with different *c*_MgTe_ values, some with stronger and some with weaker correlations. A model taking into account interactions over more than the nearest-neighbor can be ruled out, since such models produce valleys of different shapes (Welberry, 2010 ▸).

**Figure 10:**
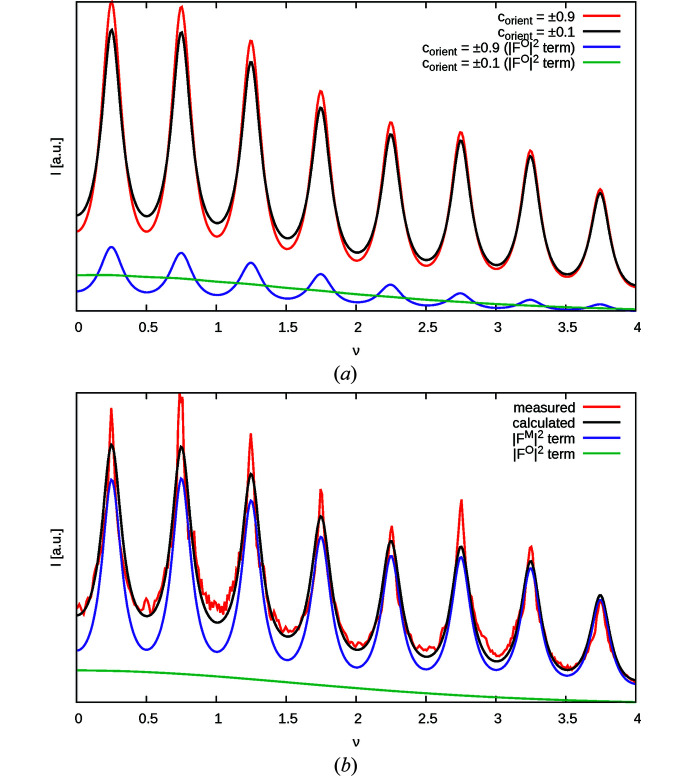
Intensity *I*(12ν) of the 12ν rod with fixed *c*_MgTe_ = −0.338 (*a*) calculated for |*c*_orient_| = 0.9 and |*c*_orient_| = 0.0 and (*b*) with |*c*_orient_| = 0 optimized against experimental data. Intensity is absolute. Additionally, the contributions of (*a*) the 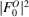 term and (*b*) the 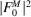 and 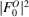 terms are shown.

Given *c*_MgTe_, |*c*_orient_| can be determined from the *h* + *k* odd rods with neither *h* nor *k* divisible by three. Fig. 10 ▸(*a*) gives *I*(12ν) plots with *c*_MgTe_ = −0.338 derived from the 10ν rod and |*c*_orient_| = 0, 0.9, where the contribution of the 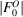 term is shown separately. The 12ν rod features the highest relative contribution of the O3 atom to the scattering intensity (§3.6). Even on this rod and with the extreme values of |*c*_orient_|, the effect on the peak shape is rather subtle.

Independent LS optimization with fixed *c*_MgTe_ yielded a zero correlation of |*c*_orient_| for all rods listed in Table 7 ▸. The refinement of the 12ν rod is displayed in Fig. 10 ▸(*b*). Again, without convolution of the experimental peak broadening slightly improved residuals are obtained. However, in both cases a zero *c*_orient_ is derived. We conclude that the orientation of the [MgO_6_] and [TeO_6_] octahedra is mostly random, which means that the problem of identical diffraction for pairs of structures becomes a moot point, since the sign of *c*_orient_ ≈ 0 is irrelevant.

### 3.12. Diffuse scattering on *h* + *k* even rods   

As has been argued above, pairs of structures with *c*_orient_ of the same absolute value produce the same diffraction intensity on *h* + *k* odd rods. Moreover, under the idealization of *d*(Te—O3) = *d*(Mg—O3) the *h* + *k* even rods are identical for all stacking arrangements. Thus, such pairs of idealized structures can be considered as homometric.

**Figure 11:**
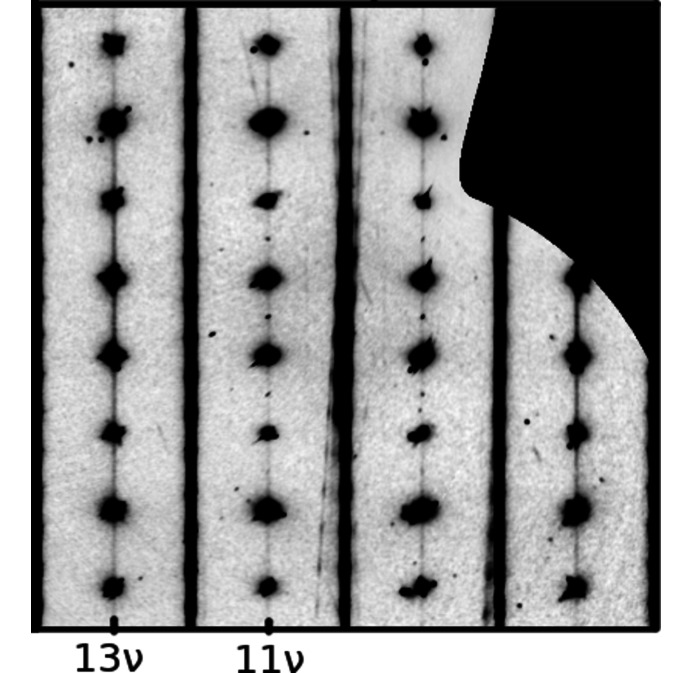
1*k* *l* layer with intensities scaled to make weakest effects visible.

Since these assumptions are not perfectly realized, very weak diffuse scattering is likewise observed on rods *h* + *k* even (Fig. 11 ▸). In principle, this could be used to determine the sign of *c*_orient_.

The diffuse scattering on these rods can be derived in analogy to §3.10. However, for *h* + *k* even the equalities exp[2πi(*h*/2+*k*/2+Δ*n*ν)] = exp[2πiΔ*n*ν] and exp[2πi(*h*/2+Δ*n*ν)] = exp[2πi(*k*/2+Δ*n*ν)] hold, since *h* and *k* are either both even or both odd. Conveniently, these terms can be generalized to exp[2πi(Δ*n* *h*/2+Δ*n*ν)] for even and odd Δ*n* (see §3.6). Factoring out the probabilities of equation (29), the leading factors in the equations analogous to equations (31) and (32) are 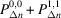 = 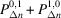 = 1. Ultimately, the general expression for *s*_Δ*n*_ on rods *h* + *k* even is

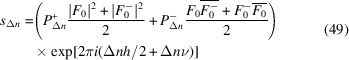

which also holds for more general growth models. Substituting the probabilities of equations (36) and (37), the intensity *I*(*hk*ν) is (setting *m* = Δ*n*)

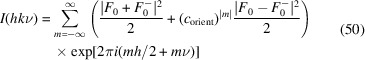




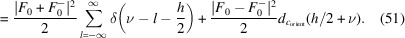

The first term corresponds to the Bragg peaks of the family structure, again committing the abuse of notation. For *h* + *k* even, equation (45) becomes 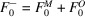 and therefore

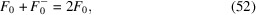




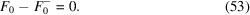

Thus, as shown in §3.6, under the assumption *d*(Te—O3) = *d*(Mg—O3), only family reflections are observed on rods *h* + *k* even. Non-equal Te—O and Mg—O distances lead to a non-vanishing 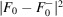 and thus diffuse scattering as described in the second term of equation (51). For distinctly positive *c*_orient_ one would expect additional peaks on top of the family reflections and valleys between the family reflections. For negative *c*_orient_, additional peaks would be observed between the family reflections. This is consistent with the MDO_1_ and MDO_2_ polytypes: the lattice of the former (*c*_orient_ = 1) does not allow for reflections between family reflections owing to the *B*-centering, whereas the latter (*c*_orient_ = −1) has a primitive Bravais lattice and features systematic non-space group absences in the idealized case, which should be observable for noticeable deviations therefrom.

**Figure 12:**
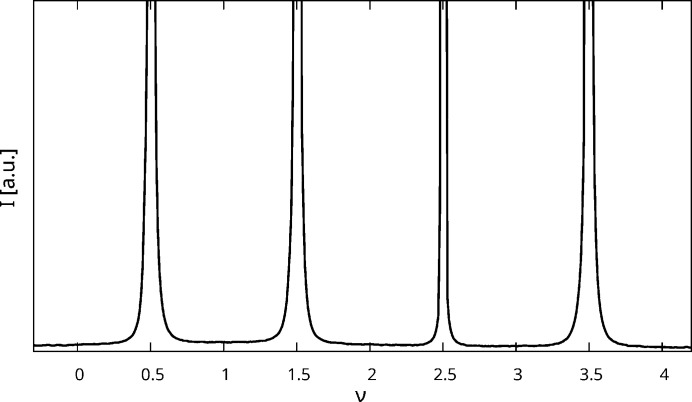
11ν rod extracted from synchrotron data. The intensity was scaled up to show the minute diffuse scattering base line. Intensities are absolute.

In the actual diffraction pattern (Fig. 11 ▸), the minute streaks are basically structureless, confirming the low correlation *c*_orient_ ≈ 0. Tiny sharp spots are observed, which can however be explained by λ/2 radiation. To prove this assignment, a Mg(H_2_O)_2_[TeO_2_(OH)_4_] crystal was quickly scanned using synchrotron radiation, which confirmed the structureless diffuse scattering on rods *h* + *k* even (Fig. 12 ▸).

It has to be noted that equation (51) does not allow for a simple quantitative estimation of the diffuse scattering in cases where *c*_orient_ ≠ 0, because the |*F*_0_ − 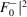 factor does not only represent the deviation of the equidistance of O3 from Mg and Te, but also generally the deviation from the idealized *p*4/*m* symmetry, which certainly exists as shown by enlarged ADPs. Moreover, the origin difference between adjacent layers might deviate slightly from the ideal Δ**o** = **a**/2 + **c**_0_/2 or Δ**o** = **b**/2 + **c**_0_/2, which would likewise invalidate the reasoning in §3.5 and lead to faint diffuse scattering.

All these deviations from the idealized model can not be simply derived from single-crystal experiments, since they will differ depending on the adjacent layers. Owing to missing structural data of all MDO polytypes, these would have to be derived by relaxation, for example with DFT methods.

In any case, this is of no concern here, since there appears to be no significant *c*_orient_.

In summary, we propose a model with a negative correlation of the Mg/Te stacking *c*_MgTe_ ≈ −0.34 and a *c*_orient_ ≈ 0 correlation of the orientation, with the caveat that the peak shape is not described perfectly, as the crystals might be composed of domains with varying *c*_MgTe_.

### 3.13. Rearrangement of the crystal structure over time   

**Figure 13:**
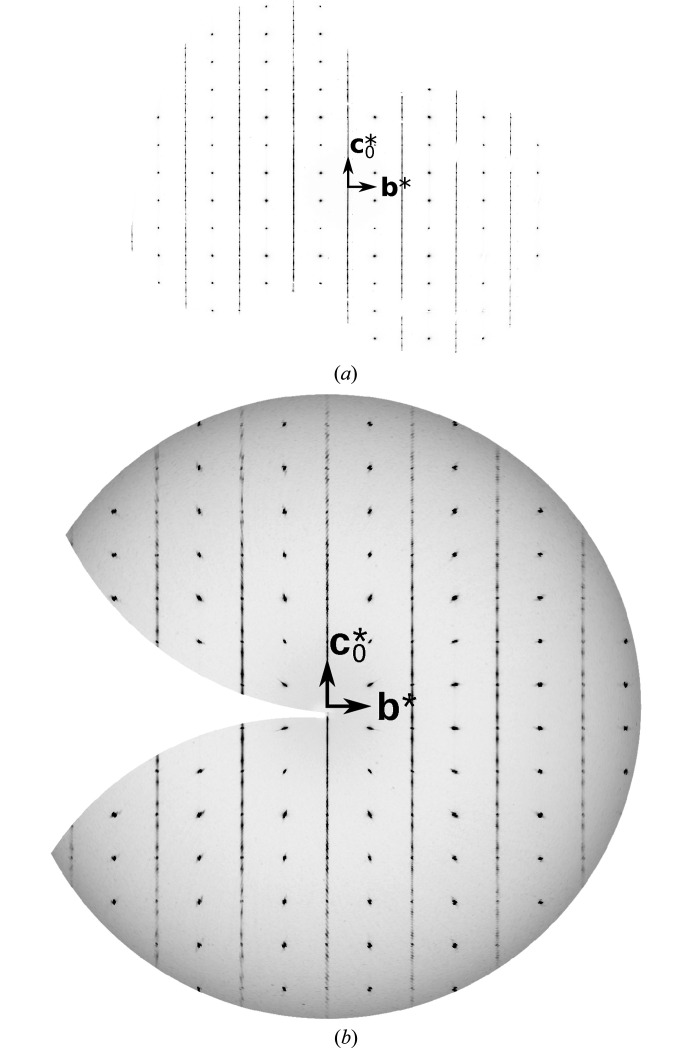
1*k*ν plane of crystals seasoned for six years collected (*a*) at the X06DA beamline and (*b*) the IPDS in-house system.

The synchrotron measurement described in the previous section was performed on a newly isolated crystal seasoned for six years at 15–35°C in a closed glass vial containing residual gel from the synthesis. Much to our surprise, in this experiment additional sharp characteristic reflections were observed on rods *h* + *k* odd at integer and half-integer ν-values [Fig. 13 ▸(*a*)], as would be expected for MDO_1/2_ polytypes. To confirm the appearance of ordered domains, a different crystal was measured in-house and likewise featured sharp reflections with *h* + *k* odd [Fig. 13 ▸(*b*)], though only half as many.

**Figure 14:**
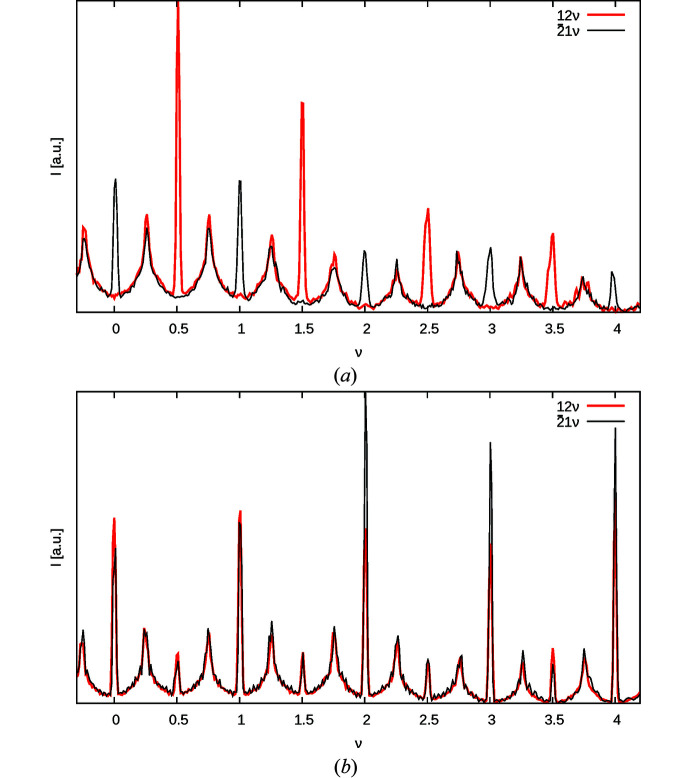
Comparison of the 12ν and 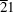 ν rods of crystals seasoned for six years measured at (*a*) the IPDS in-house system and (*b*) the X06DA beamline.

For ordered MDO_1/2_ polytypes (*c*_MgTe_ = 1), the simplifications of §3.10 do not apply and therefore macroscopic MDO_1_ and MDO_2_ polytypes produce distinctly different intensities on rods *h* + *k* odd. The sharp reflections of the second crystal can be indexed with the *B*-centered cell of the MDO_1_ polytype [*h* + 2ν even, Fig. 14 ▸(*a*)]. The location of the sharp reflections of the first crystals could in principle be explained by the MDO_2_ polytype. According to structure factor calculations, for MDO_2_ one would expect alternately strong and weak characteristic reflections at opposite positions on 12ν and 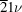 rods. In the actual crystal though, the strong and weak characteristic reflections appear at the same ν-values [Fig. 14 ▸(*b*)], which means that the characteristic reflections are probably due to two MDO_1_ orientation states, related by a fourfold rotation.

**Figure 15:**
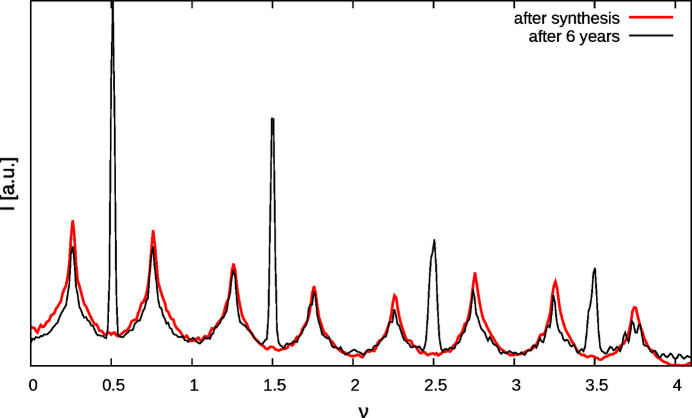
Comparison of the 12ν rods of a freshly synthesized crystal and a crystal seasoned for six years, both measured at the IPDS in-house system.

The shape of the diffuse scattering essentially stays the same during seasoning of the crystals (Fig. 15 ▸). We conclude that the disordered domains slowly convert to ordered MDO_1_ polytypes.

We recently measured three newly isolated crystals of the same synthesis batch after an additional one year time-period and all of them clearly contained ordered MDO_1_ fragments. One of the crystals was twinned by fourfold rotation. The amount of diffuse scattering did not decrease significantly compared to the previous year. Thus, the kinetics and preconditions of the transition are not yet understood.

### 3.14. Thermal behavior   

**Figure 16:**
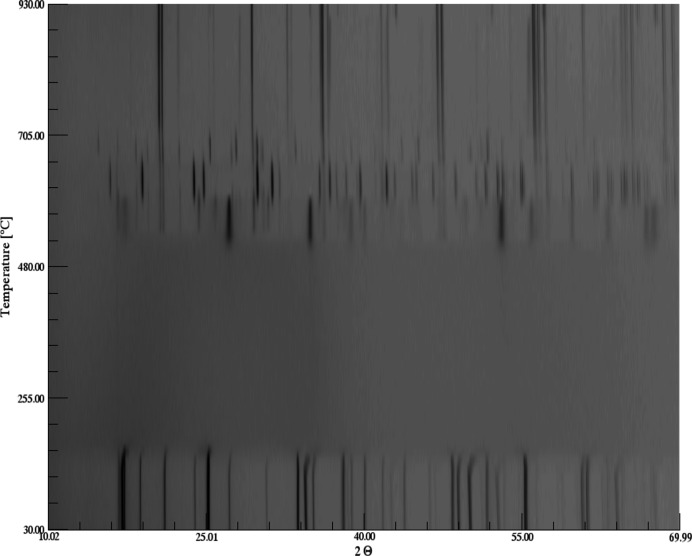
Temperature-dependent X-ray diffraction pattern of Mg(H_2_O)_2_[TeO_2_(OH)_4_].

**Figure 17:**
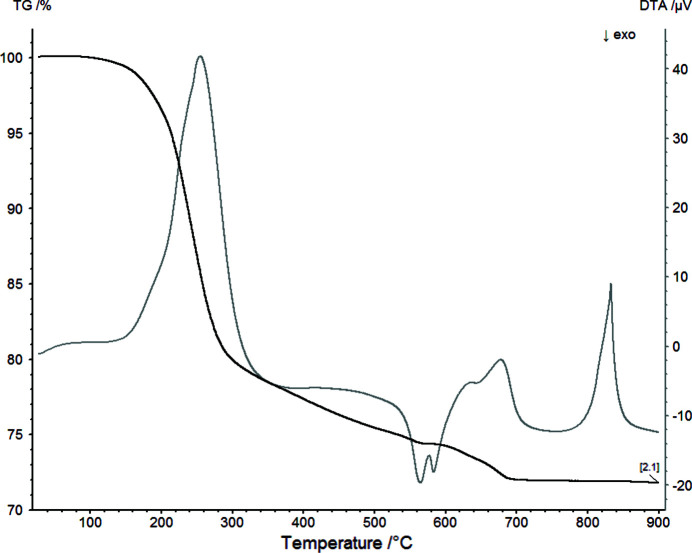
STA measurement of Mg(H_2_O)_2_[TeO_2_(OH)_4_] with the TG curve in black and the DTA curve in grey.

The thermal decomposition of Mg(H_2_O)_2_[TeO_2_(OH)_4_] is connected with a multi-step mechanism between 30–900°C. The hydrous phase is stable up to a temperature of ∼160°C in the oven chamber (Fig. 16 ▸; PXRD), followed by an amorphization. According to the TG/DTA curves (Fig. 17 ▸; STA), the onsets of the associated mass loss and the endothermic effect due to dehydration are at ∼195°C. We ascribe the different temperatures of the Mg(H_2_O)_2_[TeO_2_(OH)_4_] stability field to the different timescales of the two measurement techniques. Whereas the temperature-dependent PXRD measurement is slow due to stepwise heating rates and long measurement times, the STA measurement is much faster with continuous heating rates and much shorter measurement times. The mass loss of ∼25% up to a temperature of 500°C is due to release of water and oxygen according to the mass spectra. The amorphous phase remains up to 510°C where first reflections appear, indicating a crystallization of new phases as shown by a two-step exothermal effect at ∼550°C, also associated with a release of small amounts of water (onset DTA first step 545°C, second step 575°C). The diffuse nature of the reflections in the stability field between ∼510°C and 570°C makes a clear assignment difficult. Besides weak reflections that could be unambiguously assigned to the formation of Mg_3_[TeO_6_], a relationship with trirutile-type Co[Sb_2_O_6_] (Reimers *et al.*, 1989 ▸) could be derived from the strong reflections. Given the very similar ionic radii for Co^2+^/Mg^2+^ and Sb^5+^/Te^6+^, respectively, this could point to possible existence of a mixed-valent Te^4+^/Te^6+^ compound with composition Mg[Te_2_O_6_]. The assumption of the existence of such a mixed-valent phase is supported by the detection of oxygen in the mass analyzer during the preceding decomposition step. The assumed mixed-valent phase transforms above 570°C into a phase for which the diffraction pattern could be related to Mg[TeO_4_] (Sleight *et al.*, 1972 ▸), which is stable until ∼660°C. Above this temperature another phase is formed for which a relation to a known phase could not be made. Above ∼710°C only the reflections of Mg_3_[TeO_6_] are visible, associated with another small mass loss of ∼3% in the TG curve under further release of oxygen. Above this temperature no further mass loss is observed until 900°C. We currently cannot interpret the significant endothermal effect in the DTA curve in this temperature interval (onset 824°C). Since no further mass loss is observed here, this effect could be related either to a structural phase transition of (parts of) the remaining material or to a melting of an amorphous content thereof. Both effects cannot be related with the temperature-dependent diffraction pattern, *e.g.* by splitting or vanishing of reflections or a significantly broader background. It should be noted that the same material heated up to 1000°C in another experiment similar to the STA study resulted in the complete formation of a glass.

In summary, the decomposition mechanism of Mg(H_2_O)_2_[TeO_2_(OH)_4_] can be formulated as:

I. Mg(H_2_O)_2_[TeO_2_(OH)_4_]_(s)_ → amorphous material_(s)_ + H_2_O_(g)_, O_(g)_

II. Amorphous material_(s)_ → ‘Mg[Te_2_O_6_]’_(s)_+ Mg_3_[TeO_6_]_(s)_ + H_2_O_(g)_

IIIa. ‘Mg[Te_2_O_6_]’_(s)_ + Mg_3_[TeO_6_]_(s)_ → Mg[TeO_4_]_(s)_ + Mg_3_[TeO_6_]_(s)_

IIIb. Mg[TeO_4_]_(s)_ + Mg_3_[TeO_6_]_(s)_ → unknown phase_(s)_ + Mg_3_[TeO_6_]_(s)_

IV. Unknown phase_(s)_ + Mg_3_[TeO_6_]_(s)_ → Mg_3_[TeO_6_]_(s)_ + O_2(g)_

## 4. Conclusion and outlook   

The correlated disorder of Mg(H_2_O)_2_[TeO_2_(OH)_4_] is notable because it can be decomposed into two modes, which can be treated separately. It demonstrates the difficulties of a quantitative structure determination inherent to data sets with a significant diffuse scattering. In such cases, refinements against Bragg reflections are not sufficient and information on correlated disorder has to be derived from diffuse scattering. But even such descriptions can be ambiguous. Here, pairs of entirely different structures with opposite sign of *c*_orient_ produce virtually indistinguishable diffraction patterns. Using a small degree of idealization, simple analytical expressions describing the diffuse scattering can be derived, which allow for extremely fast calculations and a more thorough insight on the observed diffraction phenomena.

A crucial feature in the polytypism of Mg(H_2_O)_2_[TeO_2_(OH)_4_] is the hydrogen-bonding network that connects adjacent layers. Its role has been ignored in this X-ray study owing to disorder and the weak scattering power of the hydrogen atoms. A study using neutron diffraction might reveal a very different picture, possibly even necessitating the introduction of a third correlation parameter and different OD-layer symmetries. 

## Supplementary Material

Crystal structure: contains datablock(s) family, mdo34, mdo3, mdo4. DOI: 10.1107/S2052520621006223/ra5097sup1.cif

Structure factors: contains datablock(s) family. DOI: 10.1107/S2052520621006223/ra5097familysup2.hkl

Structure factors: contains datablock(s) mdo3. DOI: 10.1107/S2052520621006223/ra5097mdo3sup3.hkl

Structure factors: contains datablock(s) mdo34. DOI: 10.1107/S2052520621006223/ra5097mdo34sup4.hkl

Structure factors: contains datablock(s) mdo4. DOI: 10.1107/S2052520621006223/ra5097mdo4sup5.hkl

CCDC references: 2090412, 2090413, 2090414, 2090415

## Figures and Tables

**Table 1:** Crystal data and integration details of Mg(H_2_O)_2_[TeO_2_(OH)_4_]

	Data collection 1	Data collection 2	Data collection 3
Crystal data			
Sum formula	MgTeO_8_H_8_	MgTeO_8_H_8_	MgTeO_8_H_8_
*M_r_*	486.46	486.46	486.46
Crystal system	Tetragonal	Tetragonal	Tetragonal
Crystal form	Square bipyramid	Square bipyramid	Square bipyramid
Crystal color	Colorless	Colorless	Colorless
Crystal size (mm)	0.15 × 0.15 × 0.22	0.16 × 0.16 × 0.25	0.07 × 0.07 × 0.08
			
Data collection			
Diffractometer	Bruker KAPPA APEX II	Stoe IPDS-II	X06DA beamline
Radiation type, λ (Å)	Mo K\overline{\alpha}, 0.71073	Mo K\overline{\alpha}, 0.71073 Å	0.7085
Temperature (K)	293	293	293
Data collection method	ω- and φ-scans	ω-scan	ω-scan
θ_max_(°)	39.1	29.7	30.0
No. of measured reflections	7344	^†^	^†^
*a*, *c* (Å)	5.32820 (10), 20.6725 (4)	5.334 (2), 20.808 (5)	5.316 (2), 20.791 (4)
*V* (Å^3^)	586.886 (11)	592 (3)	587 (3)
*D_x_* (Mg m^−3^)	3.166	†	†
μ (mm^−1^)	5.167	†	†
Absorption correction	Multi-scan (*SADABS*)	†	†
*T*_min_, *T*_max_	0.32, 0.46	†	†
*R*_int_ (Laue class)	0.040 (4/*mmm*)	†	†

† No data reduction or refinement performed.

**Table 2:** Selected interatomic distances *d* (Å) and angles (°) in Mg(H_2_O)_2_[TeO_2_(OH)_4_] The data are derived from the I\overline{4}2d refinement of §3.8.

Atoms	*d*	Atoms	Angle
Te—O1	1.972 (2) (2×)	O1—Te—O1	180
Te—O3	1.906 (2) (4×)	O1—Te—O3	90.97 (6)
Mg—O2	2.044 (3) (2×)	O3—Te—O3	90.02 (10)
Mg—O3	2.064 (2) (4×)	O3—Te—O3	178.07 (8)
O1⋯O1	2.9316 (14) (2×)	O2—Mg—O2	180
O1⋯O2	2.9024 (14) (2×)	O2—Mg—O3	90.89 (5)
O2⋯O2	2.8746 (14) (2×)	O3—Mg—O3	90.01 (9)
O3⋯O3	2.525 (3)	O3—Mg—O3	178.21 (7)

**Table 3:** Fractional coordinates, multiplicity, Wyckoff letter and site symmetry in the four MDO polytypes and the family structure of Mg(H_2_O)_2_[TeO_2_(OH)_4_] The coordinates were derived from the *I*4_1_/*a* (MDO_1_ and MDO_3_), I\overline{4}2d (MDO_2_ and MDO_4_) and family structure refinements described in §3.8.

Atom	*x*	*y*	*z*	Multiplicity, Wyckoff letter, site symmetry
MDO_1_ (*B*112/*m*, **c** = 2**c**_0_)				
Te	0	0	0	2, *a*, 2/*m*
Mg	½	½	0	2, *d*, 2/*m*
O1	0	0	0.19082	2, *g*, ..2
O2	½	½	0.19786	2, *g*, ..2
O3	−0.1563	−0.3211	0	4, *i*, ..*m*
O3′	−0.3211	0.1563	0	4, *i*, ..*m*
MDO_2_ (*Pcnm*, **c** = 2**c**_0_)				
Te	0	0	0	2, *a*, \overline{1}
Mg	½	½	0	2, *c*, \overline{1}
O1	0	0	0.19074	4, *e*, ..2
O2	½	½	0.19780	4, *f*, ..2
O3	−0.1563	−0.3214	0	4, *h*, ..*m*
O3′	−0.3214	0.1563	0	4, *h*, ..*m*
MDO_3_ (*I*4_1_/*a*, **c** = 4**c**_0_)				
Te	0	0	0	4, *a*, \overline{4}
Mg	½	½	0	4, *b*, \overline{4}
O1	0	0	0.09541	8, *e*, 2..
O2	½	½	0.09893	8, *e*, 2..
O3	−0.3211	−0.1563	−0.0020	16, *f*, 1
MDO_4_ (I\overline{4}2d, **c** = 4**c**_0_)				
Te	0	0	0	4, *a*, \overline{4}
Mg	½	½	0	4, *b*, \overline{4}
O1	0	0	0.09537	8, *c*, 2..
O2	½	½	0.09890	8, *c*, 2..
O3	−0.3214	−0.1563	−0.0015	16, *e*, 1
Family (*F*4/*mmm*, **c** = 2**c**_0_)				
Te/Mg	0	0	0	2, *a*, 4/*mmm*
O1/O2	0	0	0.19445	4, *e*, 4*mm*
O3	0.3318	0.1682	0	8, *i*, *m*2*m*.

**Table 4:** Comparison of the refinements The MDO_3_:MDO_4_ ratio was derived from the occupancy of the O3/O3′ positions. The (MDO_1/2_):(MDO_3/4_) ratio was determined from the occupancy ratio of the Te/Mg positions.

	Family	1:1 MDO_3/4_	MDO_3_	MDO_4_
Space group	*F*4/*mmm*	*I*4_1_/*amd*	*I*4_1_/*a*	I\overline{4}2d
MDO_3_:MDO_4_	1:1	1:1	64:35 (3)	52.8:47.2 (12)
MDO_1/2_:MDO_3/4_	1:1	9.2:90.8 (12)	15.4:84.6 (8)	13.6:86.4 (6)
*R*[*F* ^2^ > 3σ(*F* ^2^)], *wR*(*F*)	0.0121, 0.0313	0.0204, 0.0782	0.0176, 0.0678	0.0166, 0.0581
*S*	1.33	1.61	1.34	1.17
Δρ_min_, Δρ_max_ (eÅ^−3^)	−0.45, 0.27	−0.90, 1.23	−0.71, 0.93	−0.70, 0.80
Coefficient of extinction (Becker & Coppens, 1974 ▸)	490 (110)	800 (200)	960 (170)	750 (140)
No. of parameters	11	24	28	27
Twin operation	–	–	*m*_[100]_	\overline{1}
Twin volume fractions	–	–	50.4:49.6 (6)	51:49 (9)

**Table 5:** Extreme growth model parameters and the corresponding polytypes. Note that the statistical stackings (*P*_MgTe_ = 0 or *P*_orient_ = 0) are strictly speaking not overlays or the family structure, but behave as such

Polytype	*P*_MgTe_	*c*_MgTe_	*P*_orient_	*c*_orient_
MDO_1_	1	1	1	1
MDO_2_	1	1	0	−1
MDO_3_	0	−1	1	1
MDO_4_	0	−1	0	−1
MDO_1/2_	1	1	½	0
MDO_3/4_	0	−1	½	0
Family structure	½	0	½	0

**Table 6:** Residuals of the concurrent refinement of the *h* + *k* even rods with *h* or *k* divisible by three

Rod	*R_p_* with convolution	*R_p_* without convolution
All	0.022	0.019
01ν	0.025	0.021
03ν	0.020	0.018
05ν	0.016	0.014
10ν	0.020	0.016
16ν	0.014	0.013
23ν	0.017	0.014
30ν	0.022	0.019
32ν	0.018	0.016
34ν	0.019	0.017
36ν	0.018	0.017
43ν	0.018	0.016

**Table 7:** Residuals of the individual refinements of the *h* + *k* even rods with *h* or *k* divisible by three

Rod	*R_p_* with convolution	*R_p_* without convolution
12ν	0.014	0.013
14ν	0.010	0.008
21ν	0.016	0.014
25ν	0.016	0.016
41ν	0.017	0.016
45ν	0.013	0.013

**Table 8:** Occupancy of O3 atoms in MDO_3/4_ superpositions Atom positions are idealized and given with respect to the MDO_3/4_ cell (**c** = 4**c**_0_).

Polytype	(*x*,*y*,0)^*T*^	(−*x*,*y*,0)^*T*^	(*x*,*y*,1\over 4)^*T*^	(−*x*,*y*,1\over 4)^*T*^
MDO_3_	1	0	1	0
MDO_4_	1	0	0	1
MDO_3/4_ (1 − *x* : *x*)	1	0	1 − *x*	*x*
MDO_3/4_ (1 − *x* : *x*) refined in I{\overline 4}2d	(1+ *x*)/2	(1 − *x*)/2	(1 − *x*)/2	(1 + *x*)/2

## Appendix A Symbols used

    ∝ : Proportional to.

≈ : Almost equal to.

σ : Variance of Gaussian distribution.

*n*, *m*, *h*, *k*, *l* : Integers.

*x* : Real.

*w*, *e* : Weighting function and exponent in weighting function.

*L*_*n*_ : Crystal-chemical layer with sequential index *n*.

,  : OD layers (  : octahedra,  : hydrogen-bonding network).

: Group of operations of the OD layer *A*_*n*_ not inverting the layer orientation.

: Index of the subgroup  of  .

**a**, **b** : Layer lattice basis vectors.

**c**_0_ : Vector perpendicular to layer plane with the length of one *L*_*n*_ layer width.

**c** : Basis vector of a specific polytype.

*a*, *b*, *c*_0_ : Lengths of the vectors **a**, **b**, **c**_0_.

*r*, *s* : Metric parameters describing the relative positions of adjacent OD layers.

**a***, **b***,  : Basis vectors of the dual basis to **a**, **b**, **c**_0_.

*h*, *k*, ν : Reciprocal coordinates with respect to (**a***, **b***,  ) (*h*, *k*: integers; ν: real).

*F*, *I* : Structure factor and intensity (*I* = |*F*|^2^) of a polytype or disordered stacking arrangement.

*s*_Δ*n*_ : Sum term in the calculation of *I*.

*F*_*n*_ : Structure factor of the *L*_*n*_ layer.

,  : Contributions of the non-O3 atoms and the O3 atom to  , respectively.

: Structure factor of the *L*_0_ layer reflected at (100).

*T*_O3_ : Displacement parameter of the O3 atom.

*f* ^O^ : Atomic form factor of O.

*d*(O1—O2) : Distance between atoms O1 and O2.

**o**_*n*_ : Origin of the *L*_*n*_ layer.

Δ**o**_*n*_ : Origin shift from the *L*_*n*_ to the *L*_*n*+1_ layer.

α_*n*_, β_*n*_ : Origin of the *L*_*n*_ layer in coordinates: Δo_*n*_ = α_*n*_ **a**/2 + β_*n*_ **b**/2.

ω_*n*_ : Orientation of the *L*_*n*_ layer (ω = 0,1).

Δα, Δβ, Δ*n* : Relative origin shift between two layers Δα**a** + Δβ**b** + Δ *n* **c**_0_ up to layer lattice translation (Δα, Δβ = 0, 1).

*P*(…) : Probability that the expression … holds.

*P*_MgTe_ : Probability that the origin shifts **o**_*n*_ and **o**_*n*+1_ are equal.

*P*_orient_ : Probability that the orientation of two adjacent layers is the same (ω_*n*_ equals ω_*n*+1_).

*c*_MgTe_, *c*_orient_ : Nearest-neighbor correlations *c*_MgTe_ = 2*P*_MgTe_ − 1 and *c*_orient_ = 2*P*_orient_ −1.

*d_c_* (*x*) : Shape of the diffuse scattering with nearest-neighbor correlation *c*.

: Probability that the origin shift of the (*L*_*n*_, *L*_*n*+Δ*n*_) layer pair is Δα**a** + Δβ**b** + Δ*n* **c**_0_.

,  : Probabilities that the *L*_*n*_ and *L*_*n*+Δ*n*_ possess the (ω_*n*_ = ω_*n*+Δ*n*_) or opposite (ω_*n*_ ≠ ω_*n*+Δ*n*_) orientation, respectively.

*R_p_* : Residuals for fitting one-dimensionally diffuse scattering:  .

## Appendix B Derivation of volume fractions from occupancies

The volume fraction of polytypes were derived from refinements using expressions of the type |2occ − 1|, where occ is the occupancy of an atom. This may seem surprising and is due to the symmetry used in the refinements.

Consider a superposition of MDO_3_ and MDO_4_, which conveniently possess the same unit-cell parameters. Both polytypes differ in the position of the O3 atoms, which will be considered up to fourfold rotation and translation. Representative O3 atoms of MDO_3_ and MDO_4_ are listed in the first and second row of Table 8 ▸, respectively. A 1 − *x* : *x* MDO_3/4_ superposition then possesses the O3 occupancies shown in the third row. However, when refined using the MDO_4_ symmetry (  ), the O3 positions equivalent in MDO_4_ are averaged, leading to the occupancies listed in the last row.

Thus, such a refinement features two O3 positions with the occupancies occ(O3) = (1± *x*)/2 which leads to *x* = |2occ(O3) − 1|. Note that an occupancy of occ(O3) = 0 likewise corresponds to MDO_4_, but with a different origin. An analoguous argument can be made for the Mg/Te sites.

## Appendix C General pair distribution probabilities

For Δ*n* even   and  =  = 0. Double application of equation (23) then gives

Repeated substitution of equation (57) into itself leads to

Substituting the initial term  = 1:

which can be expressed in terms of Δ*n* = 2*m* as

An analoguous reasoning applies to Δ*n* odd, though with the initial terms  =  = ½.

## Appendix D Derivation of the peak shape induced by nearest-neighbor growth models

The shape of the diffuse scattering due to nearest-neighbor correlated stacking arrangements is long known [see Welberry (2010) ▸, and references therein]. It will be briefly derived here for a general nearest-neighbor correlation *c* using geometric series.

 designates the real part.
